# The immune checkpoint molecule B7-H4 regulates β-cell mass and insulin secretion by modulating cholesterol metabolism through Stat5 signalling

**DOI:** 10.1016/j.molmet.2024.102069

**Published:** 2024-11-19

**Authors:** Fangzhen Xia, Ziteng Zhang, Zhen Qian, Xiaoyu Fang, Junxue Wang, Yan Wang, Guoting Sun, Yuefeng Yu, Ninjian Wang, Junke Zhen, Yan Liu, Yingli Lu

**Affiliations:** 1 Institute and Department of Endocrinology and Metabolism, Shanghai Ninth People's Hospital, Shanghai JiaoTong University School of Medicine, Shanghai, 200011, China; 2 iHuman Institute, ShanghaiTech University, Shanghai 201210, China; 3 Key Laboratory of Cell Differentiation and Apoptosis of Chinese Ministry of Education, Shanghai Jiao Tong University School of Medicine, Shanghai, 200025, China

**Keywords:** B7-H4, Insulin secretion, Cholesterol metabolism, Apof, Type 2 diabetes

## Abstract

**Objective:**

B7-H4 (B7S1, B7x, VTCN1) is an important immune checkpoint molecule that maintains immune homeostasis and is also expressed in pancreatic β cells. The polymorphism of B7-H4 influences the prevalence of Type 2 diabetes (T2D), suggesting a potential role of B7-H4 in the physiological function of pancreatic β cells and the pathogenesis of T2D.

**Methods:**

β-cell-specific B7-H4 knockout mice (B7-H4 cKO mice) and their wild-type littermates were used to investigate the *in vivo* effects of B7-H4 on pancreatic β-cell morphology and function. AAV2/8-ins2-B7H4 and a control virus were infused via the pancreatic intraduct into high-fat diet (HFD)-treated mice to elucidate the therapeutic effect of B7-H4. RNA sequencing was conducted on primary islets. A Luminex assay was used to quantify cytokine changes in B7-H4 cKO mice. Electron microscopy imaging was used to observe insulin secretory vesicles in pancreatic β cells.

**Results:**

Lesion of B7-H4 in β cells results in glucose intolerance due to reduced β-cell mass and deficient insulin secretion, whereas overexpression of B7-H4 in β cells ameliorates glucose intolerance in HFD-fed mice. Mechanistically, B7-H4 deficiency activates signal transducer and activator of transcription 5 (Stat5) signalling, which inhibits the expression of apolipoprotein F (Apof), leading to reduced cholesterol efflux and accumulated cholesterol in β cells, thereby impairing insulin processing and secretion. Overexpression of Apof in β cells or intraperitoneal injection of a Stat5 inhibitor reverses the metabolic phenotype and insulin secretion deficiency in B7-H4 cKO mice.

**Conclusion:**

Our study demonstrated that B7-H4 plays an important role in regulating β-cell mass and insulin secretion, which may shed new light on the development of novel strategies for T2D treatment.

## Highlights

• The expression of B7-H4 was evaluated in β cells from HFD-induced obese and genetically modified T2D mice.
• Lesions of B7-H4 in β cells result in glucose intolerance due to a reduced β-cell mass and deficient insulin secretion.
• B7-H4 regulates insulin secretion by modulating cholesterol metabolism through Stat5 signaling-regulated Apof expression.
• B7-H4 is an important linker for β cells to maintain islet immune homeostasis and their normal functions.

## Graphical abstract

**Figure:**
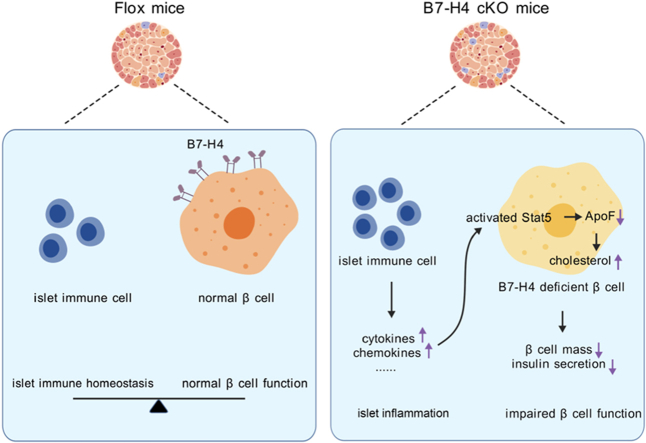

## 1 Introduction

Type 2 diabetes (T2D) accounts for approximately 90% of the 537 million diabetes cases worldwide and has become one of the most serious public health problems, with increasing morbidity and mortality [1]. Islet inflammation is believed to be a hallmark of diabetes and is involved in β-cell dysfunction in patients with T2D [2,3]. Infiltrated cells, molecules and cytokines in the islet microenvironment are critical for islet inflammation. Therapies targeting cytokines have yielded encouraging results [4,5], but molecular targets are still rare. To develop therapies that target the fundamental pathological processes of T2D, new molecular targets and therapeutic strategies are needed.

Immune checkpoint molecules play important roles in regulating immune responses to maintain immune homeostasis. Some immune checkpoint molecules are also expressed in human islet β cells and play metabolic roles in regulating insulin secretion [[6], [7], [8], [9]]. Moreover, anticancer therapies that block certain immune checkpoint molecules can lead to immune checkpoint inhibitor-associated diabetes (ICI-DM), which goes beyond autoimmune insulin-dependent diabetes [10,11]. Moreover, glucolipotoxicity initiates β-cell death through certain immune checkpoint pathways [12]. These studies suggest that immune checkpoint molecules may play important roles in the physiological function of pancreatic β cells and even in the pathogenesis of T2D. We previously explored the differentially expressed immune checkpoint genes in the pancreas of T2D patients and healthy controls and identified six immune checkpoint genes, including CD44, CD47, SIRPA, HAVCR2, TNFSF9, and VTCN1 [13], that might link islet inflammation and β-cell dysfunction in the pathological processes of T2D.

B7-H4 (B7x, B7S1 and VTCN1), the protein encoded by the VTCN1 gene, is a member of the B7 family of proteins expressed on the surfaces of antigen-presenting cells (APCs) and negatively regulates the activities of T cells [14,15]. B7-H4 is also highly expressed in numerous tumour tissues and is positively correlated with tumour growth and metastasis due to evasion of immune surveillance [16]. Studies from our laboratory and many other groups have shown that B7-H4 has a direct effect on tumorigenesis by regulating the cellar behaviour of tumour cells [17,18]. B7-H4 has been reported to contribute to maternal–foetal immune tolerance [19]. It is also important for immune homeostasis in pancreatic islets. The reduction in B7-H4 expression on APCs leads to the loss of peripheral protection in pancreatic islets and is associated with the development of autoimmune diabetes, whereas early treatment of NOD mice with B7-H4 reduces the incidence of autoimmune diabetes [[20], [21], [22], [23], [24]]. Because B7-H4 is also expressed in pancreatic islet cells and colocalizes with insulin [25], B7-H4 may play a physiological role in the function of β cells. Moreover, the polymorphism of B7-H4 influences the prevalence of T2D [26], suggesting that B7-H4 may play important roles in the pathogenesis of T2D.

The production and secretion of insulin is an important function of pancreatic β cells and involves complex processes that include the synthesis of preproinsulin, trans-Golgi network sorting of proinsulin, the formation of immature secretory vesicles, the maturation of insulin secretory vesicles, the transport of insulin secretory vesicles and the exocytosis of insulin vesicles at the plasma membrane [27]. Cholesterol, an important component of the phospholipid bilayer of the cell membrane, is critical for the maintenance of the β-cell membrane structure and is thus essential for glucose-stimulated insulin secretion. Alterations in the cholesterol content of β cells lead to enlarged insulin secretory vesicles and reduced glucose-stimulated insulin secretion [[28], [29], [30], [31]]. Cholesterol homeostasis is also important for immune cell functions. Apolipoprotein L, a specific and minor component of HDL, is a conserved gene family involved in innate immunity. APOL genes have recently been reported as novel regulators of islet inflammation and may contribute to β-cell damage during the development of diabetes [32]. These studies suggest that cholesterol metabolism may be linked to islet inflammation and β-cell dysfunction.

Signal transducer and activator of transcription 5 (Stat5, Stat5A and Stat5B in mice) is a member of the STAT transcription factor family. Stat5 is an essential regulator of cytokines or growth factors that induce cell survival and proliferation, as well as the crosstalk between STAT signalling and metabolism. Stat5 directly and indirectly regulates various metabolism-related processes in cancer cells and the metabolism of immune cells in the tumour microenvironment, playing crucial roles in the metabolic reprogramming of tumours [33]. Stat5 is also involved in metabolic disorders. Knockout of Stat5 in T cells ameliorates high-cholesterol and high-fat diet-induced hypercholesterolemia by influencing cholesterol metabolism in the liver [34]. Deletion of the liver-specific Stat5 gene alters the expression of bile acid metabolism genes and reduces liver damage in lithogenic diet-fed mice [35]. Studies on the role of Stat5 in β-cell functions are rare. Previous studies have indicated that Stat5 activity in β cells influences susceptibility to experimentally induced type 1 and type 2 diabetes [36], but the mechanism is still unclear.

In the present study, we demonstrated that the immune checkpoint molecule B7-H4 is an important molecular linker for β cells to maintain islet immune homeostasis and their normal functions, regulating β-cell mass and insulin processing and secretion. Specifically, knocking out B7-H4 in β cells results in glucose intolerance due to decreased β-cell mass and deficient insulin secretion, whereas overexpressing B7-H4 in β cells ameliorates glucose intolerance in HFD-fed mice. The role of B7-H4 in regulating insulin processing and secretion is attributed to cholesterol metabolism regulated by the Stat5 signalling pathway. B7-H4 decreases Stat5 signalling to upregulate the expression of Apof, followed by increased cholesterol outflow and glucose-induced insulin secretion. These findings provide new information for the development of strategies to treat T2D.

## 2 Materials and methods

### 2.1 Mice

C57BL/6J genetic background B7-H4 fl/fl mice, in which exon 2 of the Vtcn1 gene is flanked by two loxP sites, were generated by Cyagen Biosciences (Suzhou, China) with CRISPER/Cas9 system. B6.Cg-Tg(Ins2-cre)25Mgn/J, commonly called Rip-Cre mice in which Cre recombinase is under the control of the rat insulin II promoter and B6.FVB-Tg(Pdx1-cre)6Tuv/J, commonly called Pdx-Cre mice in which Cre recombinase is under the transcriptional control of the mouse Pdx1 promoter, were purchased from the Jackson Laboratory. 8-weeks old genetic type 2 diabetic models db/db mice were purchased from Shanghai laboratory animal center. Mice homozygous for the floxed B7-H4 allele (Flox) were crossed with Rip-Cre or Pdx-Cre transgenic mice to create β-cell–specific B7-H4 knockout mice (Rip-Cre; B7-H4 fl/fl; B7-H4 cKO or Pdx-Cre; B7-H4 fl/fl; PB7-H4 cKO) and their control littermates. Genotyping was identified by PCR on genomic DNA obtained from toes. Assessment of deletion efficiency were performed by quantitative PCR on cDNA obtained from islets. To avoid interference of the Cre transgene in the mouse metabolic phenotype [37], we used Cre and Flox mice as littermate controls. All animal experiments were approved by the Laboratory Animal Ethics Committee in Ninth People's Hospital Affiliated to Shanghai Jiao Tong University School of Medicine (Approval No.: SH9H-2023-A824-1), according to the Guide for the Care and Use of Laboratory Animals prepared by the National Academy of Sciences and published by the National Institutes of Health.

The mice were housed in groups of 4–6 per cage under standard housing conditions with a temperature-controlled (22 °C ± 2 °C) animal room on a 12-hour light/dark cycle with food and water available ad libitum. Mice were fed with a normal chow diet (NCD) or high-fat diet (HFD) (60% fat, 20% protein, and 20% carbohydrate) (catalog no. D12492; Research Diets, New Brunswick, NJ).

### 2.2 Cell line culture and high glucose, palmitate and IL-1β treatment

The murine insulinoma derived MIN6 cells were cultured in Dulbecco's Modified Eagle Medium (DMEM) (Gibco, 11965092) with 15% fetal bovine serum (Gibco, A3160801), 100 mg/mL streptomycin, 100 units/mL penicillin (Gibco, 15140-122), 10 mmol/L HEPES (Gibco, 15630-080), 1 mmol/L Sodium pyruvate (Gibco, 11360-070), and 50 μM β-Mercaptoethanol (Sigma-Aldrich, 444203) at 37 °C in 5% CO2 incubators. For high-glucose treatment, MIN6 cells were incubated in modified DMEM medium (Gibco, 11885084, contains 5.5 mM glucose) for 48h with various concentrations of glucose (low glucose: 5.5 mmol/L glucose + 19.5 mmol/L mannitol; high glucose: 25 mmol/L glucose). For palmitate and IL-1β treatment, MIN6 cells were incubated in completed DMEM medium for 48h with various concentrations of palmitate (Sigma-Aldrich, 0, 0.1, 0.2, 0.3, 0.4, 0.5 mmol/L) and recombinant mouse IL-1β (Peprotech, 0, 2, 4, 6, 8, 10 ng/mL).

### 2.3 Metabolic assays

Body weight and food intake were estimated every week from 5 weeks old to 10 weeks. For glucose tolerance tests (GTTs), mice were injected with glucose (2 g per kg of body weight for NCD-fed mice and 1 g per kg of body weight for HFD-fed mice) intraperitoneally (i.p.) after 16 h of fasting. Blood glucose levels were measured at 0,15, 30, 60, 90, and 120 min after injection from the tail vein with a glucometer monitor (Jonson), and plasma insulin levels were measured at 0, 5, 15, 30, and 60 min after injection using Highly Sensitive Mouse Insulin ELISA kit (EZassy, MS100). For insulin tolerance tests (ITTs), mice were fasted for 6 h in the morning and i.p. injected with human insulin (Novorapid, Novo Nordisk) (1unite per kg of body weight) diluted in saline solution, and measured blood glucose levels at 0, 15, 30, 60, and 90 min after injection. Blood was obtained after an overnight fast to assess the fasting glucose, insulin, glucagon, triglyceride (TG), and total cholesterol (TC). Serum TG were evaluated using Triglyceride Assay Kit (Nanjing Jiancheng, A110-1-1) and TC were measured using LabAssay Cholesterol Kit (Wako, KBR0082). Serum glucagon was evaluated using Mouse Glucogon EIL Kit (RayBio, EIAM-GLU-1).

### 2.4 Pancreatic intraductal viral infusion

To elucidate the therapeutic effect of B7-H4 on obesity associated metabolic dysfunction, 6- weeks-old Flox mice fed with 8 weeks of NCD or HFD were then injected with adeno-associated virus serotype 2/8 vector (AAV2/8)-rat insulin2 promoter (Rip)-specific B7-H4-ZSGreen (AAV2/8-ins2-B7H4) or with control virus (AAV2/8-Ctrl) (Hanbio Biotechnology Co.Ltd., Shanghai, China). To clarify the role of Apof in β cell function affected by β-cell–specific B7-H4 knockout, 6-weeks-old Flox and B7-H4 cKO mice fed with 8 weeks of HFD were injected with AAV2/8-Apof-3xFlag-eGFP or control virus (AAV2/8-eGFP) (Genomeditech, Shanghai, China).The viruses (10^12^ genome copy particles/mL) were infused at a rate of 6 μL/min by pancreatic intraductal viral infusion as described previously [38].

### 2.5 Stat5 inhibitor treatment

To ascertain whether enhanced Stat5 is involved in β cell dysfunction in B7-H4 cKO mice, 8 weeks-old B7H4 cKO mice were subsequently randomized to intraperitoneally injected with either vehicle control (VC) or pimozide, Stat5 inhibitor (Selleck, S4358, CAS: 2062-78-4, 10 mg/kg body weight daily) for 2 weeks.

### 2.6 Primary pancreatic islet isolation and culture

To isolate mouse islets, pancreata were perfused through the common bile duct with a HBSS collagenase solution (1  g/L collagenase P 33768624 and 1.5  g/L DNAse I 1014159001, Roche) and digested in the same solution in a 37 °C water bath for 25 min. After shaking for 15 s, pancreata were washed three times with HBSS supplemented with 0.5% bovine serum albumin (BSA) with centrifuge 1500*g* for 1 min. Islets were handpicked under a dissection microscope using a pipette with a 10 μL tip and pooled into a dish with DMEM (Gibco, 11885084) containing 5.5 mM glucose, 100 units/mL penicillin, 100 mg/mL streptomycin and 10% FCS. Islets were used directly for Western blot, RNA isolation or cell culture in humid environment containing 5% CO2.

### 2.7 *In vitro* glucose stimulated insulin secretion assays

For *in vitro* insulin secretion stimulation assays, 10 islets were placed in each well of the 48-well plate and cultured overnight in 300 μl complete medium and then pre-incubated for 30 min in modified Krebs-Ringer bicarbonate buffer (KRB; 115 mM NaCl, 4.7 mM KCl, 2.6 mM CaCl_2_·2(H2O), 1.2 mM KH_2_PO_4_, 1.2 mM MgSO_4_·(2H_2_O), 10 mM HEPES, 0.5% bovine serum albumin, pH 7.4) containing 2.8 mM glucose. KRB was then replaced by KRB with 2.8 mM glucose and collected after 1 h to determine the basal insulin release. This was followed by 1 h incubation in KRB with 16.7 mM glucose to determine the glucose stimulated insulin release (GSIS). After supernatant collection, islet protein content was extracted with 0.18 N hydrochloride acid in 70% ethanol to measure insulin and protein concentration (10 islets in 100 μL) with Wide Filed Mouse Insulin ELISA kit (EZassy, MS300) and BCA Kit (Solarbio, PC0020) respectively.

### 2.8 Histological analysis

For islet β cell and α cell area analysis, the pancreata (divided into three parts including the head, body and tail, three sections per animal, Figure 3A) of Flox and B7H4 cKO mice were fixed overnight in 4% paraformaldehyde at 4 °C, followed by paraffin embedding. Sections were deparaffinized, re-hydrated and incubated overnight at 4 °C with primary antibody against insulin (1:200) (Abcam, Ab181547) and glucagon (1:200) (Cell Signaling Technology, 2760S), followed by detection with FITC- (1:1,000) (Abcam, Ab150077) and Cy3-conjugated secondary antibody (1:500) (Abcam, Ab6939) respectively, counterstained with DAPI and mounted with Mounting medium (Dako). Pancreas sections were analyzed using a Hamamatsu flash 4.0 camera with a 4× Nikon Objective NA 0.2 and automatically scanned using a Nikon NiE with Prior PL-200 slide loading robot. Insulin+ and glucagon + area and distribution were calculated with NIS-Elements software (Nikon). Anti-B7-H4(1:200) (Abcam, Ab108336), anti-CD31(1:400) (Abcam, Ab182981), anti-insulin, anti-glucagon and anti-F4/80 (1:400) (Abcam, Ab6640) were used for detection of islet B7-H4 expression and location, Anti-Cleaved Caspase-3(1:400) (Cell Signaling Technology, 9661) and anti-insulin were used for detection of β cell apoptosis. All images in TIFF format were analyzed in ImageJ and Imaris (Oxford instruments) after background noise reduction and contrast adjustment. Measurements such as area, integrated density, and Feret's diameter were recorded for regions exceeding the threshold. Parameters for β cells were set based on their size and shape for cell counting. Colocalization analysis was performed to study the relationship between two antigens. For quantitative analysis, regions of interest (ROIs) were selected, and the integrated density was measured. Data obtained from these measurements were exported and compiled in a spreadsheet or statistical software for further analysis.

### 2.9 Luminex assay

Serum cytokines and chemokines of Flox and B7H4 cKO mice were quantified by the high-performance Luminex 31-plex method (Bio-Rad Laboratories, lot number 64469922). Test items included cytokines (IFN-γ, IL-1β, IL-2, IL-4, IL-6, IL-10, IL-16, and TNF-α), chemokines (CCL1, CCL2, CCL3, CCL4, CCL5, CCL7, CCL11, CCL12, CCL17, CCL19, CCL20, CCL22, CCL24, CCL27, CXCL1, CXCL1, CXCL5, CXCL10, CXCL11, CXCL12, CXCL13, CXCL16 and CX3CL1).

### 2.10 RNA extraction and qRT-PCR

Total RNA was extracted using RNeasy Mini Kit (QIAGEN, 74104) and RNAiso Plus (Takara, 9108) for islets and MIN6 cells. cDNA was prepared with PrimeScript™ RT Master Mix (Takara, RR036A) according to manufacturer's instructions. Gene expression was determined with PowerUp™ SYBR™ Green and the realtime PCR system QuantStudio 6 Pro (Applied Biosystems). Data were quantified using the comparative 2-▵▵CT method using β-actin as a normalization control. The primer sequences are shown in Supplementary Table 1.

### 2.11 RNA sequencing

Total RNA was extracted from islets of Flox mice, B7H4 cKO mice and HFD-fed Flox mice, HFD-fed Flox with B7H4 overexpression mice for RNA-sequencing library construction. The RNA libraries were sequenced on the illumina NovaseqTM 6000 platform by OE Biotech, Inc., Shanghai, China. Gene expression levels are represented as fragments per kilobase per million mapped reads (FPKM). The raw sequence data have been submitted to the NCBI Gene Expression Omnibus (GEO) datasets with accession number GSE268688.

### 2.12 Bioinformatics analysis of the genotype tissue expression (GTEx) dataset

The data used for the analyses described in this study were obtained from GTEx Portal on October13, 2023 (https://www.gtexportal.org/home/downloads/adultgtex/bulk_tissue_expression). To study how the effect of B7-H4 at the molecular level, we analyzed RNA-seq data from 328 human pancreas samples in the GTEx portal. We compared the pancreas gene expression by establishing two groups based on the B7-H4 expression level in the pancreas (highest quartile vs lowest quartile) using the R 4.2.1.

### 2.13 Cytoplasmic and nuclear protein extraction and western blot

Pancreatic islets total proteins were extracted as previously described [39]. 800 islets from each group were used to extract cytoplasmic and nuclear protein using Cytoplasmic and Nuclear Protein Extraction Kit (Solarbio,R0050). Primary antibodies were as follows: anti-B7-H4 (1:500, R&D Systems, AF2154), anti-Apof (1:250; Abcam, ab231585), anti-Stat5 (1:1,000; Cell Signaling Technology, 94205), anti-phospho-Stat5 (Tyr694) (1:1,000; Cell Signaling Technology, 4322), anti-Lamin B1 (1:5000; Proteintech, 12987-1-AP), anti-α-Tubulin (1:5000; Proteintech, HRP-66031), anti-GAPDH (1:2,500; Proteintech, 60004-1-Ig), and anti-β-actin (1:5,000; Proteintech, 66009-1-Ig).

### 2.14 Transmission electron microscopy

Islets were fixed overnight at 4 °C in 2.5% glutaraldehyde in 0.1 M phosphate buffer (pH 7.4) and then postfixed with 2 % osmium tetroxide for 1 h. Following serial alcohol dehydration (30, 50, 70, 80, 90, and 100%), the samples were embedded in epon resin, cut into 70-nm sections, and stained with 2% uranyl acetate for examination with a Zeiss GeminiSEM 460 electron microscope. Insulin vesicles were segmented by Cellpose (Version: 2.2.3) [40]. The diameters, density and distances of insuin vesicle from the cellular membrane were quantified using Image J software (Version: 2.9.0).

### 2.15 Statistical analysis

GraphPad Prism software (version 9.0.0; GraphPad, San Diego, CA, USA) was used to obtain graph presentations, curve fittings, statistics, and *P* values. For comparison of two groups, unpaired two-tailed Student's t-test was used for parametric, or Mann-Whitney test was used for non-parametric distribution. And for more than two groups, one-way ANOVA with Bonferroni's multiple comparison was used for parametrically distributed data. A *P* value < 0.05 was considered significant. Error bars in histograms indicated the standard error of the mean (SEM).

## 3 Results

### 3.1 B7-H4 expression is evaluated in diabetic pancreatic β cells

**Figure 1:**
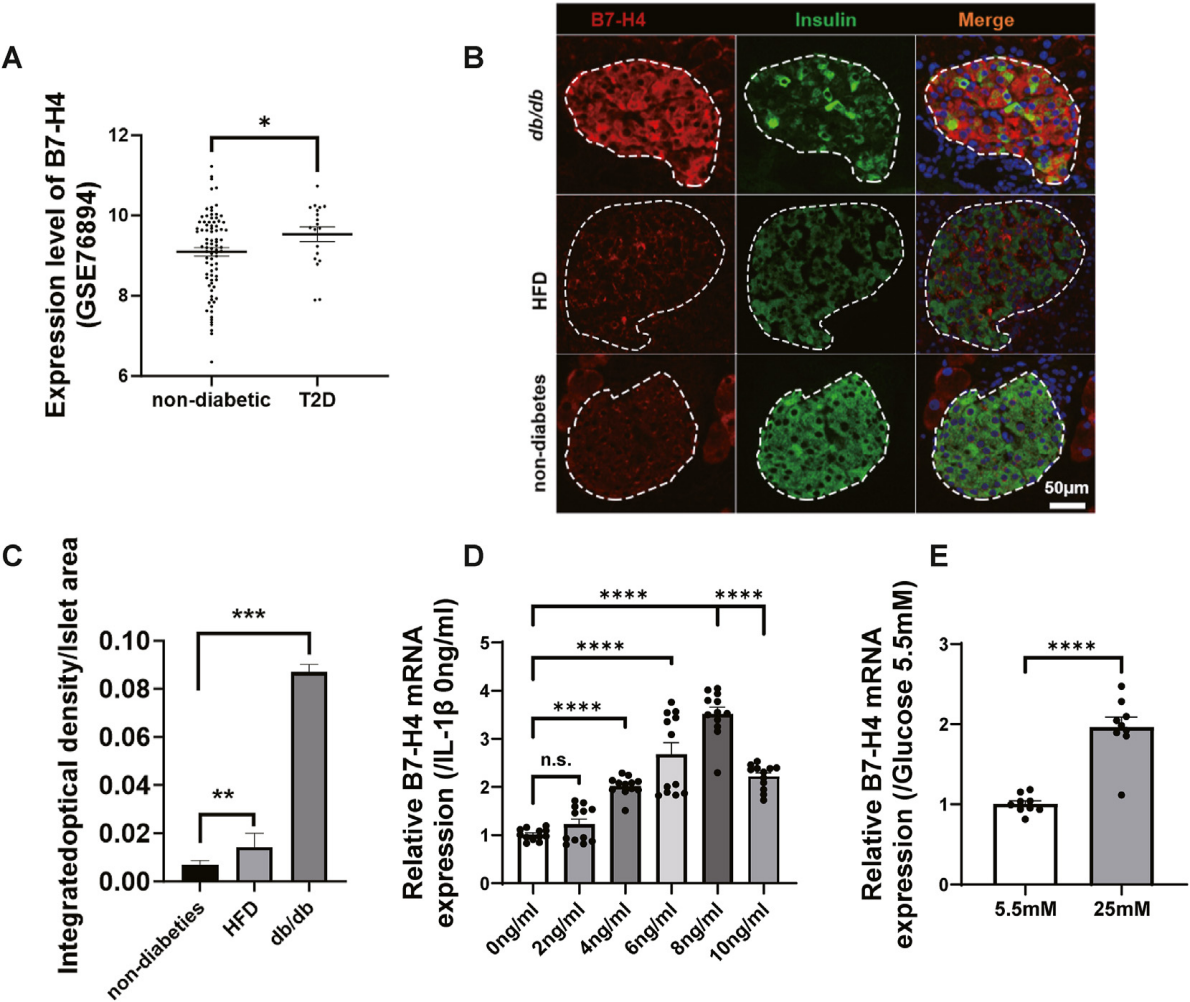
B7-H4 expression is evaluated in diabetic pancreatic β cells. (A) RNA expression of B7-H4 in pancreatic islets samples of GSE 76894 (n = 84 for non-diabetes, n = 19 for T2D). (B) Representative images of pancreatic islet immunofluorescence staining in the indicated groups. Red, B7-H4; Green, Insulin; Blue, DAPI. (C) The quantitation of B7-H4 estimated by integrated optical density/islet area in the indicated groups. non-diabetes, n = 20; HFD, n = 13; db/db, n = 15. (D) B7-H4 mRNA expression of MIN6 cell incubated in the medium with various concentrations of IL-1β for 48h. n = 12 per group. (E) B7-H4 mRNA expression of MIN6 cell incubated in the medium with various concentration of glucose for 48h. 5.5 mM, n = 9; 25 mM, n = 9. Data are shown as mean ± SEM. ∗: P < 0.05; ∗∗: P < 0.01; ∗∗∗: P < 0.001; n.s.: not significant. Mann-Whitney test was used in (A); unpaired two-tailed Student's t-test was used in (E); one-way ANOVA with Bonferroni's multiple comparison was used in (C) and (D). Three independent experiments were performed.

Identification of functional immune molecules in diabetic islet cells may be critical for developing novel strategies for T2D treatment. Immune checkpoint molecules are important molecules that maintain immune homeostasis and have also been reported to play metabolic roles in human islet β cells, suggesting potential roles in islet metabolic inflammation in T2D. Previously, we screened islet transcriptomic data from patients with T2D and healthy controls and reported that six immune checkpoint genes, including CD44, CD47, SIRPA, HAVCR2, TNFSF9, and VTCN1, were highly expressed on human diabetic islet cells, although the functions and underlying mechanisms are not clear [13]. Interestingly, one of the identified candidates, B7-H4 (B7x, B7S1 and VTCN1), the protein encoded by the VTCN1 gene, which is expressed primarily on splenic lymphocytes, was also expressed on islets, as determined by Western blot analysis (sFigure 1A). Gene expression data from the GEO database, a public functional genomics data repository, revealed that patients with T2D from the dataset of GSE76894 had increased mRNA levels of B7-H4 in pancreatic islets (Figure 1A). In obese mice induced by HFD or in genetically modified mice with T2D (db/db mice), B7-H4 was abnormally highly expressed in the islets (Figure 1B, C). To identify the cell source of elevated B7-H4 in diabetic islets, we detected the coexpression of B7-H4 with β cells (insulin), α cells (glucagon), vascular endothelial cells (CD31) and macrophages (F4/80) via two-colour immunofluorescence staining and found that B7-H4 was coexpressed mainly with insulin but not with glucagon, CD31 or F4/80 (Figure 1B and sFigure 1B), indicating that B7-H4 is expressed mainly in diabetic β cells. Diabetic pancreatic β cells are in chronic inflammatory states with hyperglycaemia and hyperlipemia. To determine the factors that regulate B7-H4 expression in diabetic β cells, glucose, palmitate and IL-1β were added to MIN6 cell cultures. We observed that high glucose and IL-1β concentrations could stimulate B7-H4 expression (Figure 1D, E), whereas palmitate had no direct effect on B7-H4 expression (sFigure 1C).

### 3.2 B7-H4 knockout in pancreatic β cells results in glucose intolerance and insulin secretion deficiency

To investigate the physiological function of B7-H4 in pancreatic β cells and whether it is involved in the pathological process of T2D, we generated β-cell-specific B7-H4 knockout (B7-H4 cKO) mice by crossing loxP-flanked B7-H4 fl/fl mice (the targeting strategy is shown in sFigure 2A) with Rip-Cre transgenic mice. The genotypes of all offspring were identified via PCR (sFigure 2B). mRNA quantification revealed that the B7-H4 level was 86% lower in islets from B7-H4 cKO mice than in those from Flox mice (sFigure 2C). Western blotting further confirmed a significant reduction in B7-H4 expression in islets from B7-H4 cKO mice (sFigure 2D).

**Figure 2:**
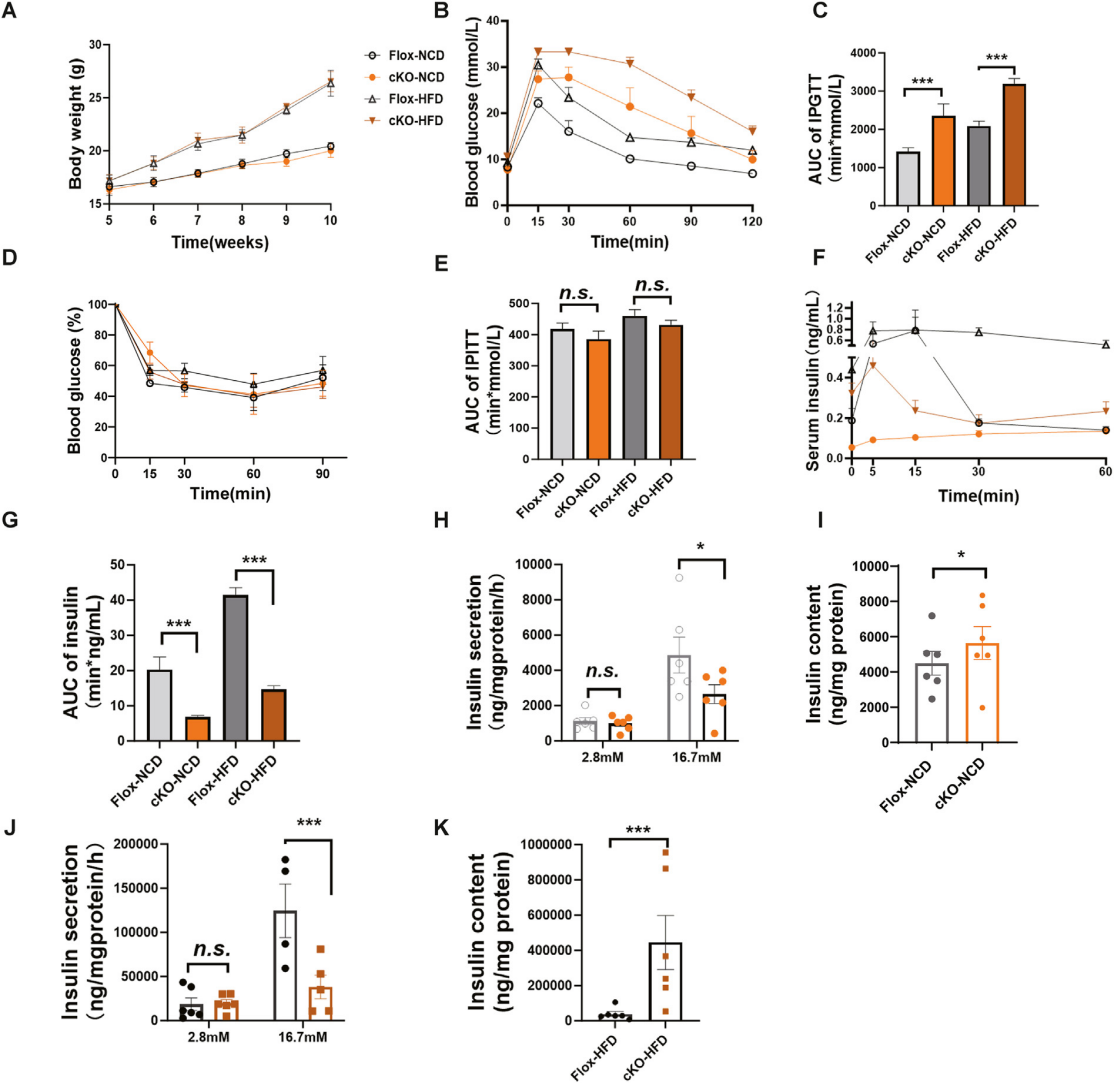
B7-H4 Knock out in pancreatic β-cells damages glucose disposal and insulin secretion. (A) Body weight of mice in the indicated groups. n = 6 per group. (B and C) GTT results of mice in the indicated groups. n = 5–8 per group. (D and E) ITT results of mice in the indicated groups. n = 6–9 per group. (F and G) In vivo GSIS results of mice in the indicated groups. n = 5–8 per group. (H and J) In vitro GSIS results in the indicated groups. (H), NCD; (J), HFD. n = 4–6 per group. (I and K) Insulin contents in the indicated groups. (I), NCD; (K), HFD. n = 6 per group. Data are shown as mean ± SEM. ∗: P < 0.05; ∗∗∗: P < 0.001; n.s.: not significant. Unpaired two-tailed Student's t-test was used in (A), (C), (E), (G–K). Three independent experiments were performed.

We fed B7-H4 cKO and littermate homozygous Flox controls a NCD or HFD, and no differences were detected between B7-H4 cKO mice and controls in terms of body weight, food intake, serum TG, total cholesterol (TC) or fasted or random blood glucose when fed a NCD (Figure 2A; sFigure 2E–I). However, glucose tolerance tests (GTTs) revealed that, in both the NCD-fed and HFD-fed B7-H4 cKO mice, glucose disposal was impaired, and B7-H4 cKO mice presented higher blood glucose levels than the controls did at 15, 30, 60, 90, and 120 min after glucose injection (Figure 2B, C). To rule out interference of the Cre transgene in glucose disposal, we conducted GTTs in Rip-Cre, Flox and B7-H4 cKO mice at 4, 8, 12 and 16 weeks of age, respectively. Glucose tolerance in Rip-Cre mice was impaired compared with that in Flox mice but was greater than that in B7-H4 cKO mice at 4 and 8 weeks of age. As time progressed, the clearance of glucose in Rip-Cre mice improved, and the blood glucose levels were slightly higher than those in Flox mice at 15 or 30 min after glucose injection at 12 and 16 weeks of age; however, in B7-H4 cKO mice, the clearance of glucose continued to be impaired, and the blood glucose levels were markedly greater than those in Flox and Rip-Cre mice at 15, 30, 60 and 90 min after glucose injection (sFigure 3A–D), suggesting that although the Cre transgene had a slight impact on glucose tolerance in the early stage, the impaired glucose tolerance in B7-H4 cKO mice was caused mainly by the specific knockout of B7-H4 in β cells. We further verified this in Pdx-1-guided β-cell-specific knockout B7-H4 mice by crossing homozygous loxP-flanked B7-H4 fl/fl mice with Pdx-Cre mice. GTT results revealed that Pdx-1-guided β-cell-specific knockout B7-H4 mice (B7-H4 fl/fl; Pdx-Cre) also presented impaired glucose clearance (sFigure 3E).

Insulin insensitivity, insulin insufficiency or elevated glucagon are the main causes of hyperglycaemia. To determine whether impaired glucose clearance in B7-H4 cKO mice is due to insulin resistance, insulin tolerance tests (ITTs) were performed in B7-H4 cKO and control mice. Both B7-H4 cKO mice and control mice exhibited normal insulin tolerance (Figure 2D, E). The concentrations of serum glucagon were comparable between these mice (sFigure 2J). Therefore, insulin insensitivity or elevated glucagon can be ruled out as a cause of hyperglycaemia upon B7-H4 knockout in β cells. To detect insulin secretion ability, glucose-induced insulin secretion (GSIS) tests were performed *in vivo*, and the results revealed that B7-H4 cKO mice had lower fasting and glucose-induced insulin release levels than did the controls in both the NCD and HFD groups and that the AUC of insulin release was significantly lower in the B7-H4 cKO mice (Figure 2F, G). In line with the *in vivo* data, the *in vitro* GSIS tests using the same number of islets isolated from B7-H4 cKO and control mice revealed lower glucose-induced insulin secretion by B7-H4 cKO islets both on a NCD (Figure 2H) and a HFD (Figure 2J). However, the insulin content was greater in islets from B7-H4 cKO mice than in those from Flox mice (Figure 2I, K). These results suggest that the inability of B7-H4 cKO mice to dispose of blood glucose is due to insulin insufficiency and secretion deficiency.

### 3.3 β-cell mass and insulin processing and secretion were impaired in B7-H4 cKO mice

**Figure 3:**
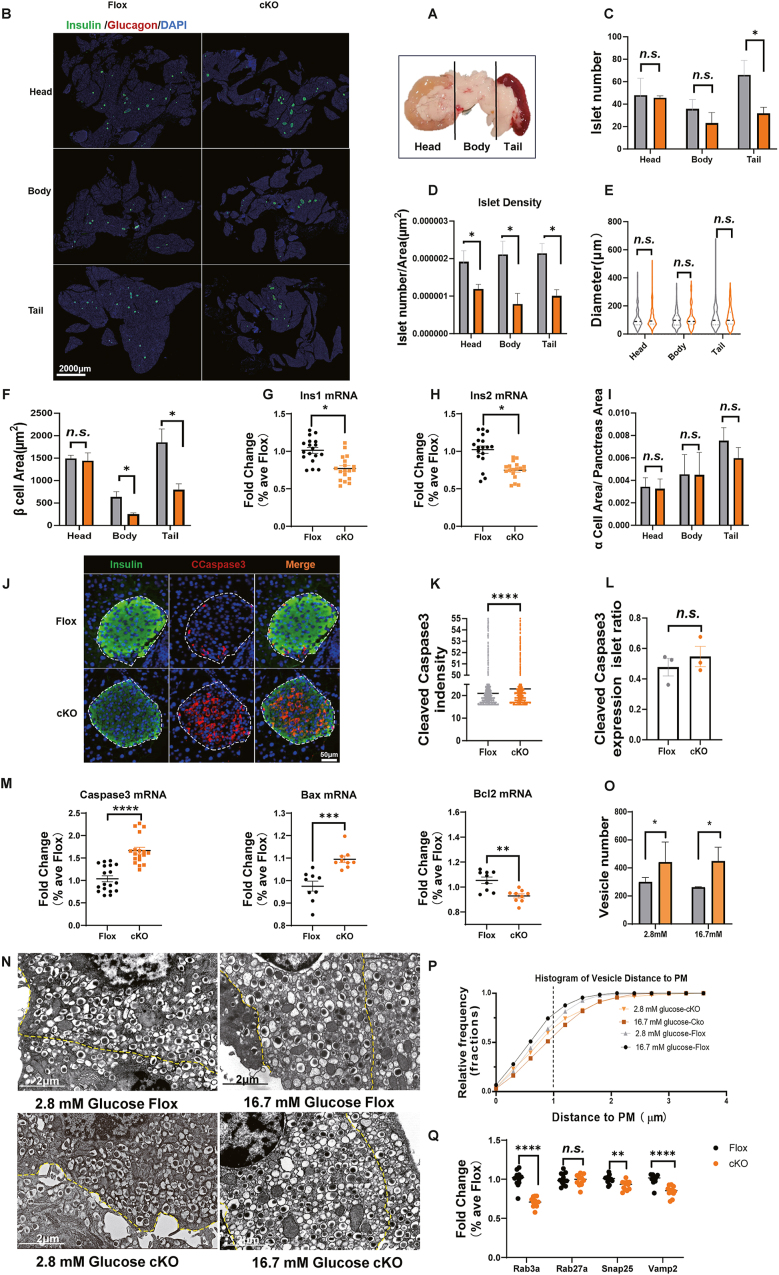
β cell mass and insulin processing and secretion were impaired in B7-H4 cKO mice. (A) Sketch map of pancreas scanning which was divided into head, body, and tail. (B) Representative images of pancreas immunofluorescence staining in the indicated groups. Green, Insulin; Red, Glucagon; Blue, DAPI. (C) Pancreatic islet number in head, body and tail of pancreas in the indicated groups. n = 3 per group. (D) The density of islet in head, body and tail of pancreas estimated by islet number/pancreas area in the indicated groups. n = 3–5 per group. (E) The size of islet in head, body and tail of pancreas in the indicated groups. n = 84–220 islets per group. (F) β cell area in head, body and tail of pancreas in the indicated groups. n = 3 per group. (G and H) mRNA expression of Ins1 and Ins2 in the indicated groups. n = 18 per group. (I) The mass of α cell estimated by α cell area/pancreas area in the indicated groups. n = 3–5 per group. (J) Representative images of pancreatic islet immunofluorescence staining in the indicated groups. Green, Insulin; red, CCaspase 3; blue, DAPI. (K) Cleaved Caspase3 intensity of islet in the indicated groups. (L) Ratio of Cleaved Caspase3 mRNA expression to pancreatic islet number. n = 3 per group. (M) Caspase 3, Bax, and Bcl mRNA expression in islets of the indicated groups. n = 9–18 per group. (N) Representative images of pancreatic islet transmission electron micrograph in the indicated groups in different concentrations of glucose. (O) Number of vesicles in β cells in the indicated groups incubated in the medium with various concentration of glucose. n = 3 per group. (P) Distance of vesicle in β cell to plasma membrane in the indicated group stimulated by various concentration of glucose. The vertical coordinate showed relative frequency of vesicle with the same distance to plasma membrane. n = 3 per group. (Q) mRNA expression of genes involved in insulin secretory granule transport and priming in islets of the indicated group. n = 12 per group. Data are shown as mean ± SEM. ∗: P < 0.05; ∗∗: P < 0.01; ∗∗∗: P < 0.001, ∗∗∗∗: P < 0.0001 n.s.: not significant. Unpaired two-tailed Student's t-test was used in (C)–(I), (K)–(M), (O), and (Q). Three independent experiments were performed.

The number and function of β cells determine insulin concentrations in the blood. To determine whether the attenuated insulin secretion in B7-H4 cKO mice is due to a decreased β-cell number, we examined the islet morphology of adult mice (12 weeks of age) by immunostaining. The whole pancreas was divided into three parts: the head, body and tail (Figure 3A). Notably, the number of islets decreased in both the body and tail of the pancreas in B7-H4 cKO mice, with a significant decrease, especially in the tail region (Figure 3B, C). The density of islets calculated by islet number/pancreatic area was also lower in B7-H4 cKO mice (Figure 3D), but the size of islets was slightly higher, although there was no significant difference between the two groups (Figure 3E), suggesting compensatory hyperplasia in B7-H4 cKO islets. The reduced insulin+ β-cell areas in B7-H4 cKO mice, especially in the body and tail of the pancreas, suggest that the hyperplasia of islets did not compensate for the loss of β-cell mass (Figure 3F). The lower mRNA expression of the Ins-1 and Ins-2 genes in islets from B7-H4 cKO mice further verified the impaired β-cell mass in B7-H4 cKO mice (Figure 3G, H). The mass of α cells was similar in B7-H4 cKO and control mice (Figure 3I). Cleaved caspase 3 is a marker of apoptosis. We used cleaved caspase 3 and insulin to double-stain the pancreas and showed that although the ratio of caspase 3^+^ islets was similar between the two groups, there was a striking increase in the expression levels of cleaved caspase 3 in the islets of B7-H4 cKO mice, suggesting that more apoptotic β cells were present in B7-H4 cKO islets (Figure 3J–L). The results of qPCR also revealed increased caspase 3 and Bax and decreased Bcl2 mRNA expression in islets from B7-H4 cKO mice (Figure 3M). These findings suggest that B7-H4 affects β-cell numbers by regulating their apoptosis.

B7-H4 cKO mice not only presented significant defects in the baseline value of insulin but the peak of insulin release also almost completely disappeared after glucose stimulation (Figure 2F), suggesting that the insufficient serum insulin in B7-H4 cKO mice was not only due to a decrease in β-cell mass. Indeed, the *in vitro* GSIS tests using the same number of islets from the two groups of mice revealed a noticeable reduction in insulin secretion but not insulin content in B7-H4 cKO islets (Figure 2H–K). Transmission electron microscopy revealed that even in a low-glycaemic environment, there were more vesicles in β cells from B7-H4 cKO mice than in those from control mice (Figure 3N–O). Moreover, the distance from vesicles to the plasma membrane was greater in B7-H4 cKO β cells than in control cells, and after stimulation with high glucose, the percentage of docked vesicles 0–2 μm apart from the plasma membrane strikingly decreased (Figure 3P), suggesting that B7-H4 knockout affected the exocytosis process of insulin secretory vesicles. qPCR results also revealed that the expression of genes involved in insulin secretory vesicle transport (Rab3a) and priming (Snap25, Vamp2) was significantly lower in islets from B7-H4 cKO mice (Figure 3Q).

### 3.4 Overexpression of B7-H4 in pancreatic β cells ameliorates glucose tolerance and improves insulin secretion

**Figure 4:**
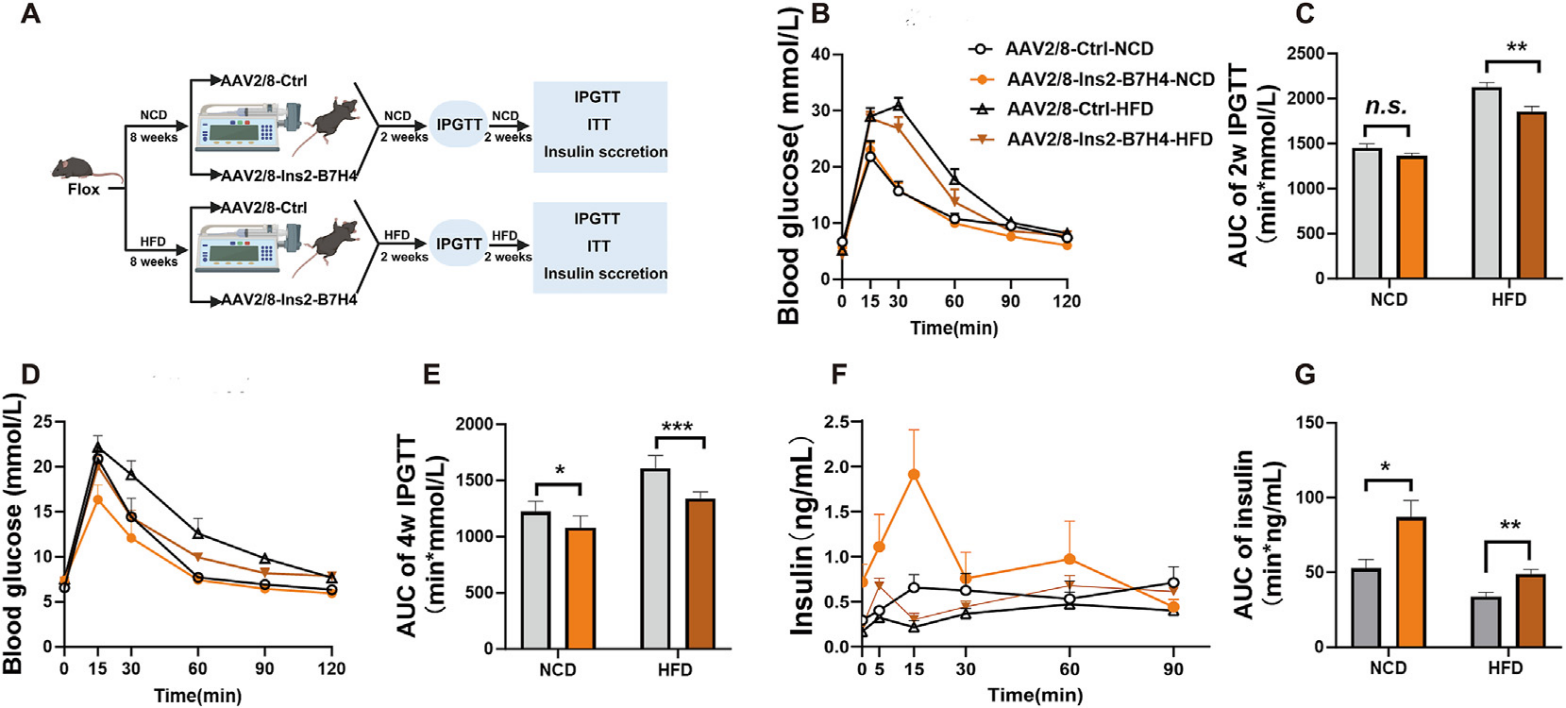
B7-H4 overexpression in pancreatic β-cells improves glucose disposal and insulin secretion. (A) Detailed treatment to B7-H4 fl/fl mice. (B and C) GTT results after 2 weeks of virus infusion in the indicated groups. n = 5–7 per group. (D and E) GTT results after 4 weeks of virus infusion in the indicated groups. n = 5–7 per group. (F and G) GSIS results after 4 weeks of virus infusion in the indicated groups. n = 5–7 per group. Data are shown as mean ± SEM. ∗: P < 0.05; ∗∗: P < 0.01; ∗∗∗: P < 0.001; n.s.: not significant. Unpaired two-tailed Student's t-test was used in (C), (E), and (G). Three independent experiments were performed.

The metabolic disorders of B7-H4 cKO mice indicated that B7-H4 has important physiological functions in islets for the maintenance of β-cell mass and cell functions. We further investigated whether B7-H4 could be used as a therapeutic treatment for obesity-associated metabolic dysfunction. As shown in Figure 4A, B7-H4 fl/fl mice were fed a HFD for 8 weeks and then infused with AAV2/8-Ins2-B7-H4 or control virus from the pancreatic intraduct to specifically overexpress B7-H4 in β cells. As the virus carries the ZSGreen fluorescent protein, islets isolated from mice after 5 weeks of virus infusion presented strong green fluorescence (sFigure 4A), and B7-H4 mRNA and protein levels were significantly elevated in islets from mice infused with AAV8-Ins2-B7-H4 compared with those from mice infused with control virus (sFigure 4B, C). To assess the effects of excess B7-H4 on the functions of healthy β cells, we also treated healthy mice fed a NCD with the same amount of virus. Two weeks after virus infusion, HFD-fed mice exhibited marked improvement in glucose clearance. Blood glucose levels were significantly lower in HFD-fed mice with β-cell-specific B7-H4 overexpression than in HFD-fed control mice at 30, 60, and 90 min after glucose injection (Figure 4B). The AUC of the IPGTT also revealed ameliorated glucose tolerance in β-cell-specific B7-H4-overexpressing HFD-fed mice (Figure 4C). However, there was no significant effect on healthy mice fed a NCD (Figure 4B, C). After 2 more weeks, β-cell-specific B7-H4 overexpression significantly improved glucose clearance in both HFD-fed and NCD-fed mice (Figure 4D, E), and insulin secretion improved markedly. Serum insulin concentrations increased significantly in B7-H4-overexpressing mice at every time point after glucose stimulation, even during fasting (Figure 4F, G). However, there were no significant changes in insulin sensitivity detected by ITTs (sFigure 4D, E). Thus, β-cell-specific B7-H4 overexpression has robust effects on insulin secretion and glucose tolerance, indicating that B7-H4 may be an ideal molecular target for improving β-cell function.

### 3.5 RNA-seq of islets indicates that B7-H4 changes the expression of β-cell function and insulin secretion markers

**Figure 5:**
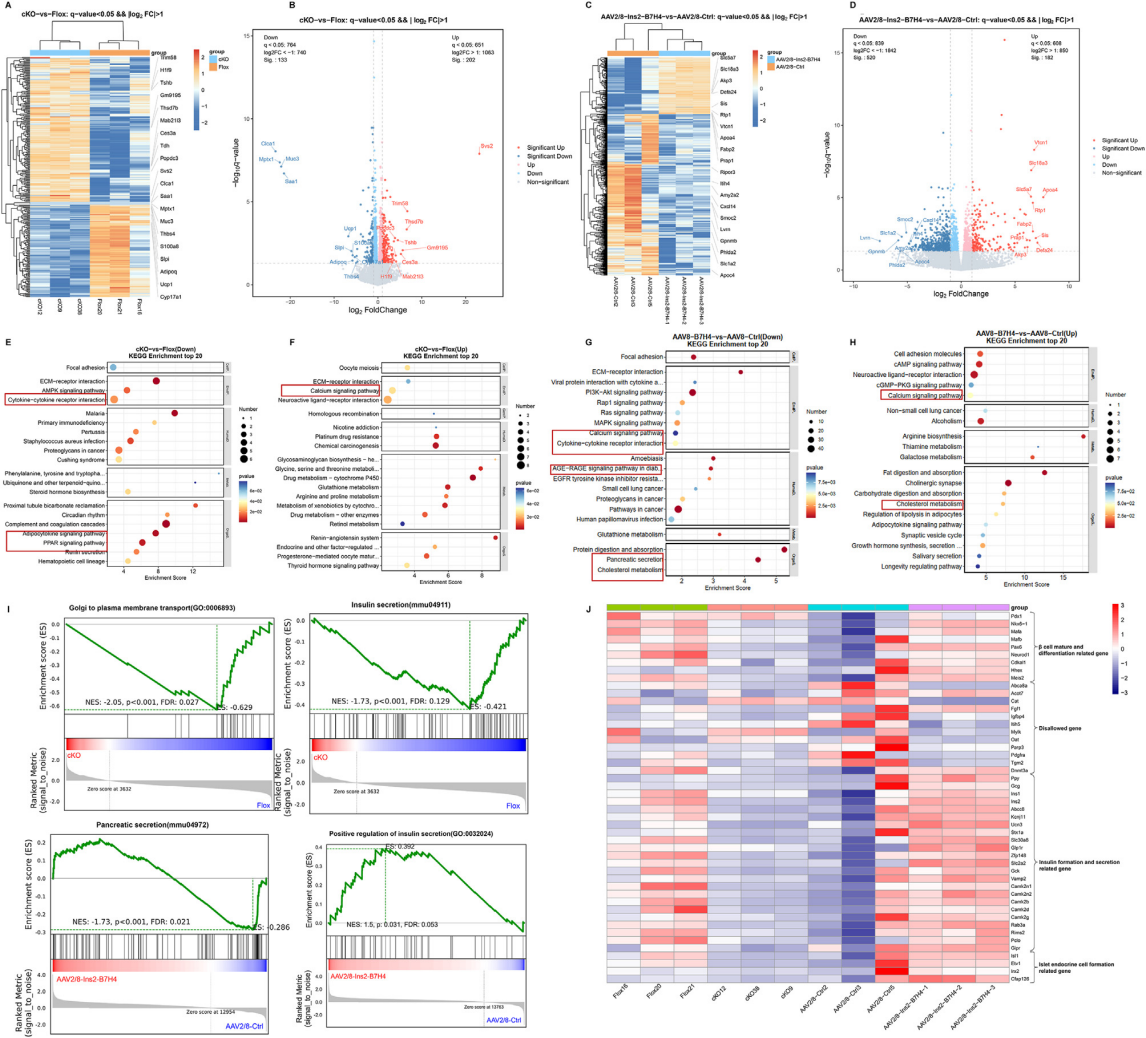
RNA-seq on islets indicates B7-H4 changed the expression of β cell function and insulin secretion markers. (A and B) Heatmap and volcano plot displaying differentially expressed genes between Flox (n = 3) and cKO (n = 3). (C and D) Heatmap and volcano plot displaying differentially expressed genes in AAV2/8-Ins2-B7H4 (n = 3) and AAV2/8-Ctrl (n = 3). (E and F) KEGG pathway enrichment analysis of differentially expressed genes in Flox (n = 3) and cKO (n = 3), which showed that downregulated genes enriched in cytokine–cytokine receptor interaction, adipocytokine signaling pathway and PPAR signaling pathway, and upregulated genes enriched in calcium signaling pathway and retinol metabolism. (G and H) KEGG pathway enrichment analysis of differentially expressed genes in AAV2/8-Ins2-B7H4 (n = 3) and AAV2/8-Ctrl (n = 3), which showed that DEGs in AAV2/8-Ins2-B7-H4 and AAV2/8-Ctrl mice were enriched in cytokine–cytokine receptor interaction, adipocytokine signaling pathway, AGE-RAGE signaling pathway in diabetes, calcium signaling pathway and cholesterol metabolism. (I) GSEA showed a downregulation in the Golgi to plasma membrane transport and insulin secretion pathways in B7-H4 cKO mice compared with Flox mice, and an upregulation in the pancreatic secretion and positive regulation of insulin secretion pathways in AAV2/8-Ins2-B7H4 mice compared with AAV2/8-Ctrl mice. n = 3 per group. (J) Expression heatmap of β cell mature and differentiation related genes, disallowed genes, and insulin formation and secretion related genes in the indicated groups. n = 3 per group.

To further explore the molecular mechanisms underlying the protective effects of B7-H4, we isolated pancreatic islets from B7-H4 cKO and Flox mice fed a NCD and from HFD-fed obese mice after 5 weeks of AAV2/8-Ins2-B7-H4 or AAV2/8-Ctrl infusion. Total RNA was extracted from these islets, and unbiased transcriptomic profiling was performed. Differential expression analysis with an absolute log2-fold change (FC) over 1 and a q value below 0.05 revealed 202 upregulated and 133 downregulated genes from B7-H4 cKO and Flox mice (Figure 5A, B) and 182 upregulated and 520 downregulated genes from AAV2/8-Ins2-B7-H4 and AAV2/8-Ctrl mice whose Vtcn1 (B7-H4) gene was dramatically upregulated (Figure 5C, D). Kyoto Encyclopedia of Genes and Genomes (KEGG) enrichment analysis of the differentially expressed genes (DEGs) in B7-H4 cKO and Flox mice revealed that the downregulated genes were enriched in cytokine–cytokine receptor interactions, the adipocytokine signalling pathway and the PPAR signalling pathway, and the upregulated genes were enriched in the calcium signalling pathway and retinol metabolism (Figure 5E, F). DEGs in the AAV2/8-Ins2-B7-H4 and AAV2/8-Ctrl mice were also enriched in cytokine–cytokine receptor interactions, the adipocytokine signalling pathway, the AGE‒RAGE signalling pathway in diabetes, the calcium signalling pathway and cholesterol metabolism (Figure 5G, H). Gene Ontology (GO) analysis revealed significant enrichment in several biological processes, cellular components, and molecular functions. Most notable among these genes were cell adhesin, potassium ion transport, and cholesterol efflux, which were downregulated in B7-H4 cKO mice and upregulated in B7-H4-overexpressing mice (sFigure 5A, B). Gene set enrichment analysis (GSEA) revealed downregulation of the Golgi-to-plasma membrane transport and insulin secretion pathways in B7-H4 cKO mice and upregulation of pancreatic secretion and positive regulation of insulin secretion pathways in B7-H4-overexpressing mice (Figure 5I).

To gain further insight into the effects of B7-H4 on β-cell function and insulin secretion, we analysed four groups of transcriptomic data together. Overall, the gene expression pattern of B7-H4 cKO mice was similar to that of HFD-fed mice injected with the AAV2/8-Ctrl virus, whereas HFD-fed mice that specifically overexpressed B7-H4 in β cells presented similar gene expression patterns to those of Flox mice. Markers of β-cell maturation, insulin formation- and secretion-related genes, and islet endocrine cell formation-related genes were significantly downregulated in B7-H4 cKO mice and HFD-fed obese mice. B7-H4 overexpression rescued the expression of these genes in HFD-fed obese mice. Although the expression of β-cell disallowed genes, which are typically repressed in mature adult β cells, was not changed in B7-H4 cKO mice, B7-H4 overexpression rescued the highly expressed disallowed genes caused by metabolic stress in HFD-induced obesity (Figure 5J). Taken together, the results of RNA-seq of islets indicate that B7-H4 alters the expression of β-cell function and insulin secretion markers.

### 3.6 B7-H4 maintains β-cell function and insulin secretion by regulating islet cholesterol metabolism

**Figure 6:**
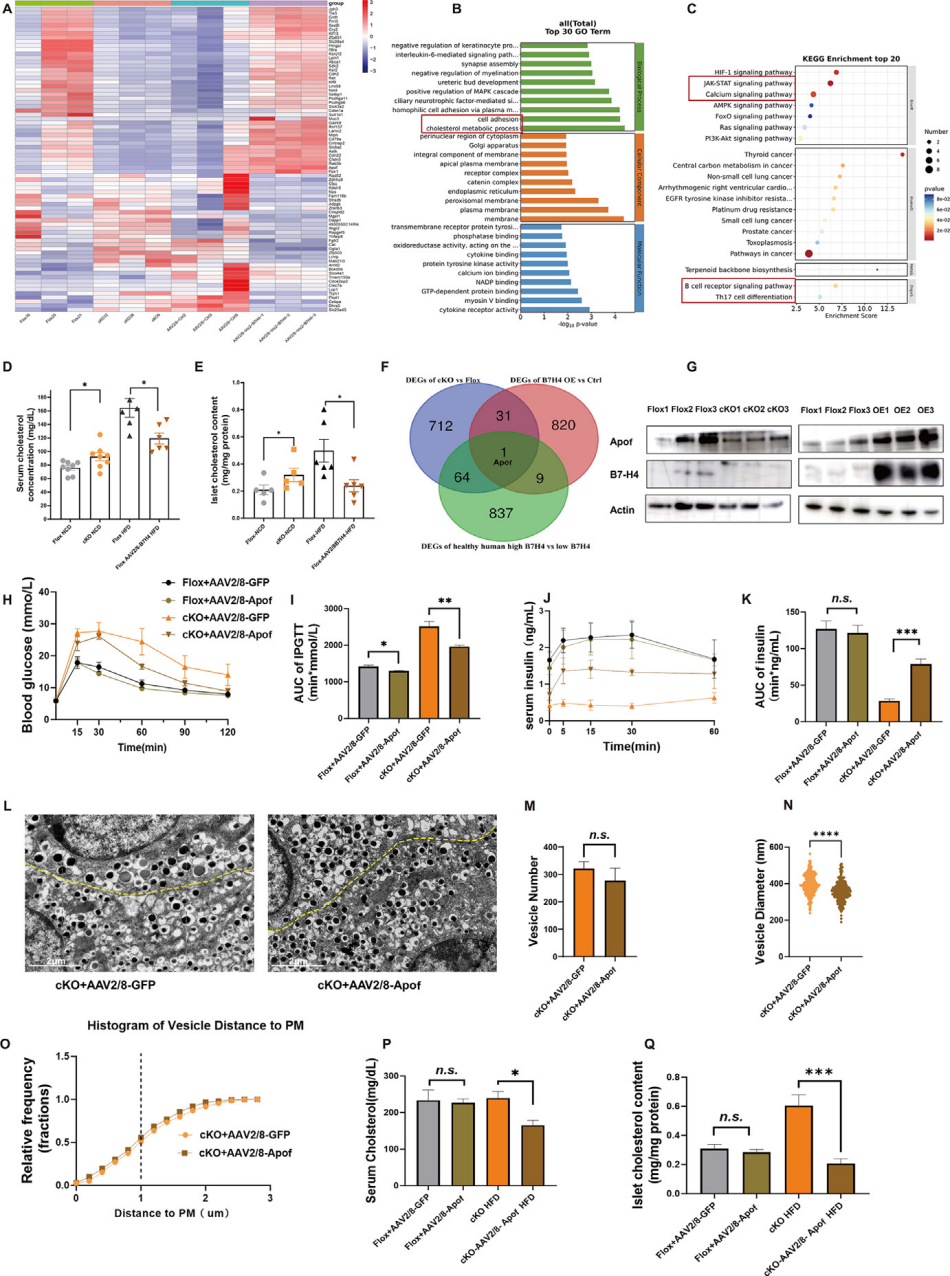
B7-H4 maintains β cell function and insulin secretion by regulating islet cholesterol metabolism. (A) Heatmap of the identified 75 genes which were significantly altered in B7-H4 cKO mice but were reversed by overexpression of B7-H4. n = 3 per group. (B and C) GO and KEGG analysis of the identified genes, which showed a significant enrichment in cell adhesion and cholesterol metabolic process, and molecular functions enriched in cytokine receptor activity, and in JAK-STAT signaling pathway and calcium signaling pathway. (D and E) Serum cholesterol concentration and pancreatic islet cholesterol content in the indicated groups. n = 4–8 per group. (F) Venn diagram showed that Apof is the only common gene regulated by B7-H4 in both humans and mice. (G) Western blot analysis of Apof expression in pancreatic islets from the indicated group, which showed that Apof in islets was downregulated by B7-H4 KO and upregulated by B7-H4 overexpression. Flox: Mice homozygous for the floxed B7-H4 allele; cKO: β-cell–specific B7-H4 knockout mice; OE: Flox mice overexpressed B7-H4 through injecting with AAV2/8-Ins2-B7H4; Apof: Apolipoprotein F; B7-H4: B7 family member, H4. (H and I) GTT results of mice in the indicated groups. n = 4–5 per group. (J and K) GSIS results of mice in the indicated groups. n = 4–5 per group. (L) Transmission electron micrograph showed that Apof overexpression made vesicles in of β cell closer to the plasma membrane. n = 3 per group. scale bar, 2um. n = 3 mouse per group. (M and N) Number and diameter of β cell vesicle in the indicated group. n = 3 mouse per group. (O) Distance of vesicle in β cell to plasma membrane in the indicated group. The vertical coordinate showed relative frequency of vesicle with the same distance to plasma membrane. n = 3 mouse per group. (P and Q) Serum cholesterol and islet cholesterol in the indicated group. n = 9–12 per group. Data are shown as mean ± SEM. ∗: P < 0.05; ∗∗: P < 0.01; ∗∗∗: P < 0.001; ∗∗∗∗: P < 0.0001; n.s.: not significant. Unpaired two-tailed Student's t-test was used in (D), (E), (I), (K), (M), (N), (P), and (Q). Three independent experiments were performed.

To better investigate the molecular mechanisms by which B7-H4 regulates β-cell function and insulin secretion, we analysed the DEGs of B7-H4 cKO and B7-H4-overexpressing islets jointly. There were 75 genes whose expression was significantly altered in B7-H4 cKO mice but whose expression was reversed by the overexpression of B7-H4 (Figure 6A). GO analysis of these genes revealed significant enrichment in cell adhesion and cholesterol metabolic processes and molecular functions enriched in cytokine receptor activity (Figure 6B). KEGG enrichment analysis revealed enrichment of the JAK-STAT signalling pathway and calcium signalling pathway (Figure 6C).

Previous studies have reported that the cholesterol content in β cells affects the amount of insulin and the size of vesicles. We detected the serum cholesterol concentration and islet cholesterol content of B7-H4 cKO mice and B7-H4-overexpressing mice. Interestingly, we found that the serum cholesterol concentration was low, but the islet cholesterol content was significantly higher in B7-H4 cKO mice, whereas B7-H4 overexpression strongly reduced the serum and islet cholesterol concentrations in HFD-fed mice (Figure 6D, E). To study how B7-H4 regulates the cholesterol content in β cells at the molecular level, we analysed RNA-seq data from human pancreas samples in the GTEx portal. GO and KEGG analyses revealed that the DEGs associated with high (top 25%) and low (bottom 25%) B7-H4 expression were enriched in cytokine–cytokine receptor interactions and in lipid, atherosclerosis and chemokine signalling pathways (sFigure 6A, B). We compared the 75 genes in mice whose expression was altered by B7-H4 knockout but reversed by B7-H4 overexpression with the differential genes in healthy humans with high and low expressions of B7-H4 in the GTEx database and found that Apof is a common gene regulated by B7-H4 in both humans and mice (Figure 6F). We verified that the protein levels of Apof in islets were decreased by B7-H4 KO and increased by B7-H4 overexpression via Western blotting (Figure 6G).

Apof has been reported to reduce cholesterol levels [41,42]. To study whether B7-H4 regulates insulin secretion through Apof, we overexpressed Apof in B7-H4 cKO mice via pancreatic intraductal infusion with AAV2/8-Apof- 3xFlag or AAV2/8-GFP control virus. As shown in sFigure 6C, the overexpression of Apof led to a sharp increase in the Apof protein level. Glucose tolerance was markedly improved by Apof overexpression in B7-H4 cKO mice, and the GTT results revealed a sharp reduction in blood glucose levels in B7-H4 cKO mice with Apof overexpression at 15, 30, 60, 90 and 120 min after glucose injection, although there was a slight decrease in glucose levels in Flox mice (Figure 6H, I). Insulin secretion was also markedly improved in Apof-overexpressing B7-H4 cKO mice, and serum insulin concentrations increased significantly at every time point after glucose stimulation, even during fasting, suggesting that Apof ameliorates the basal and glucose-stimulated insulin secretion deficiency induced by B7-H4 defects (Figure 6J, K). Transmission electron micrographs revealed that Apof overexpression in B7-H4 cKO mice reduced the number of retained vesicles in β cells, and these vesicles were smaller and closer to the plasma membrane (Figure 6L–O), indicating that Apof promotes the secretion of insulin secretory vesicles and improves the functional status of β cells in B7-H4 cKO mice. ITTs revealed slight changes in insulin sensitivity after Apof overexpression in B7-H4 cKO mice (sFigure 6D, E). Furthermore, we found that Apof significantly reduced the elevated serum and islet cholesterol levels in B7-H4 cKO mice (Figure 6P, Q).

### 3.7 B7-H4 regulates islet cholesterol metabolism through Stat5 signalling

**Figure 7:**
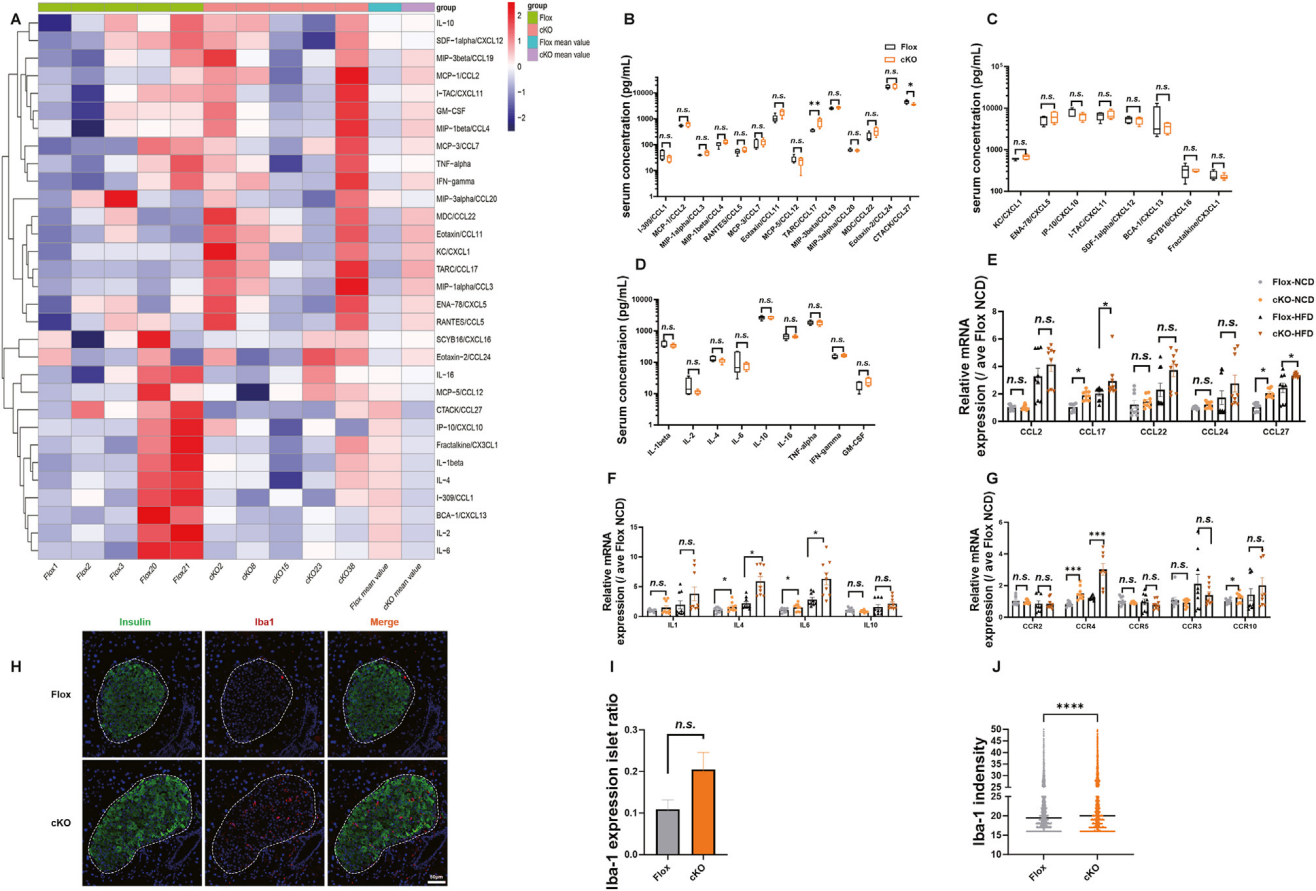
B7-H4 regulates the expression of islet cytokines and chemokine receptors. (A) Heatmap of serum concentrations of cytokines assessed by luminex multiple-factor assays in the indicated groups. n = 5 per group. (B–D) Serum concentrations of cytokines in the indicated groups. n = 5 per group. (E–G) mRNA expression of cytokines in islets from the indicated groups. n = 9 per group. (H) Representative images of pancreatic islet immunofluorescence staining in the indicated groups. Green, Insulin; Red, Iba1; Blue, DAPI. (I and J) The ratio of Iba1-positive islets (I) and Iba-1 indensity (J) in the indicated group. n = 3 mouse per group. Data are shown as mean ± SEM. ∗: P < 0.05; ∗∗∗∗: P < 0.0001; n.s.: not significant. Unpaired two-tailed Student's t-test was used in (B)–(G). Three independent experiments were performed.

In addition to β-cell function and insulin secretion markers, cytokine–cytokine receptor interactions were also enriched in DEGs of B7-H4 cKO and B7-H4-overexpressing mice, and 75 genes whose expression was altered by B7-H4 KO but reversed by B7-H4 overexpression were enriched in the JAK‒STAT signalling pathway. Previous studies have shown that B7-H4 can regulate cytokine expression in T cells [43]. These findings indicate that B7-H4 may be involved in the regulation of the immune microenvironment of islets. We performed Luminex multiple-factor assays to measure the serum concentrations of cytokines, including interleukins, CC- and CXC- chemokines and interferon, in Flox and B7-H4 cKO mice. A heatmap of these cytokines presented individually or as the means of each group revealed that MDC/CCL22, eotaxin/CCL11, KC/CXCL1, TARC/CCL17, IFN-gamma, MIP-1alpha/CCL3 and MCP-3/CCL7 were higher and that CTACK/CCL27, IP-10/CXCL10, IL-2, I-309/CCL1, IL-1beta and IL-4 were lower in the serum of B7-H4 cKO mice (Figure 7A). Analysis of the differences between the groups revealed that TARC/CCL17 expression was significantly higher and that CTACK/CCL27 expression was significantly lower in the serum of B7-H4 cKO mice (Figure 7B–D). Real-time quantitative PCR was used to detect the expression of islet cytokines in Flox and B7-H4 cKO mice fed a NCD or HDF. Consistent with the change in serum concentrations, the mRNA expression of CCL17 in islets from cKO mice was significantly upregulated, but in contrast to the serum concentrations, the mRNA levels of CCL27, IL-4 and IL-6 in islets from cKO mice were significantly greater than those in islets from Flox mice (Figure 7E, F). Moreover, we examined the mRNA levels of chemokine receptors in the islets of the four groups of mice and found that CCR4 and CCR10 were significantly upregulated in the islets of cKO mice (Figure 7G). Changes in cytokines and chemokines may alter the composition of islet immune cells. Given that the immune cells of islets are mainly macrophages, we used the myeloid marker Iba1 to stain the islets of Flox and B7-H4 cKO mice via immunofluorescence. Although there was no significant difference in the ratio of Iba1-positive islets, the intensity of Iba1 was significantly greater in B7-H4 cKO mice than in flox mice (Figure 7H–J), indicating that the number of infiltrating myeloid cells in B7-H4 cKO islets was significantly higher.

**Figure 8:**
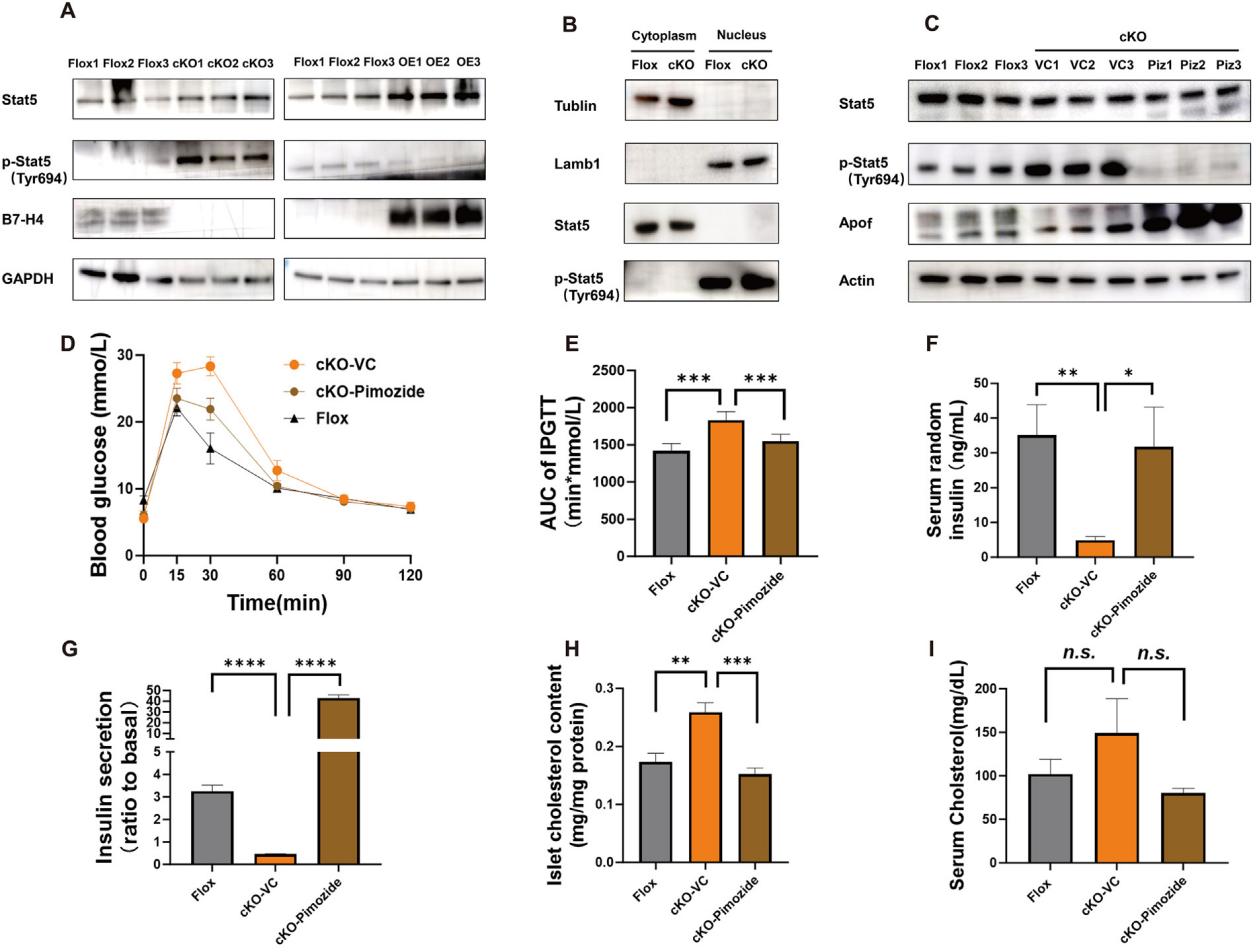
B7-H4 regulates islet cholesterol metabolism through Stat5 signaling. (A) Western blot analysis of Stat5 and phosphorylated Stat5 in islets from the indicated groups, which showed that phosphorylation levels of Stat5 were significantly increased in islets from B7-H4 cKO mice but reduced in that from B7-H4 overexpressed mice. n = 3 per group. Flox: Mice homozygous for the floxed B7-H4 allele; cKO: β-cell–specific B7-H4 knockout mice; OE: Flox mice overexpressed B7-H4 through injecting with AAV2/8-Ins2-B7H4; Stat5: Signal transducer and activator of transcription 5; p-Stat5: Phospho-signal transducer and activator of transcription 5; B7-H4: B7 family member, H4; GAPDH: Glyceraldehyde-3-Phosphate Dehydrogenase. (B) Western blot showed that phosphorylated Stat5 accumulated in the nucleus of islets from Flox and B7-H4cKO mice. Flox: Mice homozygous for the floxed B7-H4 allele; Lamb1: Laminin beta 1; Stat5: Signal transducer and activator of transcription 5; p-Stat5: Phospho-signal transducer and activator of transcription 5. (C) Western blot showed that pimozide treatment decreased the Stat5 phosphorylation levels and cholesterol content, while upregulate Apof protein levels in islets of B7-H4 cKO mice. n = 3 per group. Flox: Mice homozygous for the floxed B7-H4 allele; VC: cKO mice injected with vehicle control for 2 weeks; Piz: cKO mice injected with pimozide for 2 weeks; Stat5: Signal transducer and activator of transcription 5; p-Stat5: Phospho-signal transducer and activator of transcription 5; Apof: Apolipoprotein F. (D and E) GTT results of the mice treated by pimozide. n = 5–7 per group. (F) Serum random insulin concentration in the indicated group. n = 6 per group. (G) Results of in vitro GSIS in the indicated group. n = 6 per group. (H and I) Islet and serum cholesterol content in the indicated group. n = 6 per group. Data are shown as mean ± SEM. ∗: P < 0.05; ∗∗: P < 0.01; ∗∗∗: P < 0.001; ∗∗∗∗: P < 0.0001; n.s.: not significant. Unpaired two-tailed Student's t-test was used in (D)–(H). Three independent experiments were performed.

Cytokines that activate the JAK-STAT signalling pathway may be involved in the regulation of β-cell function and insulin secretion by B7-H4. We detected the protein and phosphorylation levels of seven members of the STAT family in islets from B7-H4 cKO and B7-H4-overexpressing mice and found that the phosphorylation levels of Stat5 were significantly higher in islets from B7-H4 cKO mice but reduced in those from B7-H4-overexpressing mice (Figure 8A). After tyrosine phosphorylation at Y694, Stat5 accumulates in the nucleus [44]. As shown in Figure 8B, phosphorylated Stat5 accumulated in the nucleus of islets from Flox and B7-H4cKO mice. To investigate whether B7-H4 regulates islet cholesterol metabolism and insulin secretion through Stat5, we treated B7-H4 cKO mice with the Stat5 inhibitor pimozide or vehicle control (VC) for 2 weeks. Pimozide treatment decreased Stat5 phosphorylation levels but increased Apof protein levels in the islets of B7-H4 cKO mice (Figure 8C). GTTs markedly improved glucose clearance in B7-H4 cKO mice treated with pimozide for two weeks, which presented significantly lower blood glucose levels at 15, 30 and 60 min after glucose injection (Figure 8D, E). The serum random insulin concentration in B7-H4 cKO mice was significantly lower than that in Flox mice, and pimozide treatment for two weeks reversed the difference in the serum insulin level between B7-H4 cKO mice and Flox mice (Figure 8F). GSIS *in vitro* revealed that pimozide treatment markedly improved glucose-stimulated insulin secretion in B7-H4 cKO mice (Figure 8G). Furthermore, we found that pimozide treatment significantly reduced the increase in islet cholesterol levels in B7-H4 cKO mice (Figure 8H), although there was no significant difference in the serum cholesterol concentration when the mice were fed a NCD; this trend was consistent with that of islet cholesterol (Figure 8I). These data indicate that B7-H4 regulates islet cholesterol metabolism to enhance β-cell function and insulin secretion through Stat5 signalling.

## 4 Discussion

Pancreatic islet inflammation is a pathological mechanism of T2D, and therapies targeting islet inflammation are promising for the prevention and treatment of T2D and its complications. However, the molecules regulating islet inflammation in the pathological process of T2D are still unclear. Through islet transcriptome data analysis of T2D patients, we identified six highly expressed immune checkpoint genes that are important for immune homeostasis. Here, we elucidate the physiological role of one of these molecules, B7-H4, in β-cell function and its pathological effects on islet inflammation and T2D.

As an inhibitory costimulatory molecule of T cells, B7-H4 has been implicated in many diseases, such as cancer, allograft rejection, and autoimmune diseases, due to its immunosuppressive function. B7-H4 is also expressed in nonhaematopoietic cells and contributes to immune tolerance, such as maternal–foetal immune tolerance [19]. In the pancreas, Jun Sik Lee et al. [20] revealed that, unlike other B7 family members, the B7-H4 protein is not expressed on immune cells but rather on pancreatic islet cells. The B7-H4 protein is expressed and colocalized with insulin [25], and its polymorphism influences the prevalence of diabetes [26]. Although it has been speculated that B7-H4 may play a potential physiological role in the function of β cells and may have pathological effects on T2D [23], there have been no relevant studies to date. For the first time, we used β-cell-specific B7-H4 knockout mice to explore the *in vivo* functions of B7-H4. We found that B7-H4 affects not only the expression of islet cytokines but also the secretion of insulin vesicles. Unlike the reduction in islet B7-H4 protein expression in T1D through the proteolytic cleavage of the B7-H4 protein from the cell membrane by the metalloproteinase nardilysin (NRD1) [21,22], we found that the expression of the islet B7-H4 protein was increased in HFD-induced obese and genetically modified T2D mice. This upregulation may be a protective mechanism against metabolic stress. Indeed, the upregulation of Nrd1 and elevated soluble B7-H4 are characteristic of patients with T1D but not T2D [22]. Immunostaining revealed that upregulated B7-H4 expression occurred mainly in islet β cells but not in pancreatic macrophages. In tumour microenvironments, cancer cells increase B7-H4 expression in response to hypoxia and transforming growth factor β1 (TGFβ1) [45,46]; IL-6 and IL-10 can also stimulate B7-H4 mRNA expression in macrophages [47]. Here, we found that in the diabetic microenvironment, high glucose and IL-1β can stimulate B7-H4 mRNA expression in the β-cell line MIN6 cells.

B7-H4 has been proposed to have a direct effect on cell functions such as the proliferation and differentiation of expressed cells [48]. Here, we revealed that B7-H4 knockout in β cells results in decreased β-cell mass and pancreatic areas in mice. Β-cell mass is determined by prenatal development of islets, postnatal compensatory growth and regeneration of β cells [49]. Dynamic changes in the β-cell mass also occur during pancreatic development and are triggered by multiple signalling pathways. Apoptosis, dedifferentiation and transdifferentiation are the main causes of reduced β-cell mass in diabetes mellitus patients. Although the decreased β-cell mass in B7-H4 cKO mice can be partially explained by an increase in β-cell apoptosis, the number of islets was significantly lower in cKO mice than in control mice, suggesting a developmental defect in β cells upon B7-H4 knockout. This might be related to altered cytokines and infiltrated immune cells in islets due to the depletion of B7-H4 in β cells. Indeed, some immune signals, such as TGF-β and IL-18, have been reported to play key roles in the development of pancreatic islets [50,51]. The altered identity of β cells has also been proposed as a mechanism for the loss of β cells in diabetes [52]. RNA-seq of islets revealed that B7-H4 knockdown in β cells downregulated the expression of β-cell maturation-related genes such as Nkx6.1, Mafa, Mafb, Pax6, and Neurod1, whereas B7-H4 overexpression rescued the expression of these genes in HFD-fed obese mice. Although the expression of β-cell disallowed genes, which are typically repressed in mature adult β cells, did not change in B7-H4 cKO mice, B7-H4 overexpression rescued the highly expressed disallowed genes caused by metabolic stress in HFD-induced obesity. These findings suggest that B7-H4 may play a promising role in reversing β-cell trans-differentiation and maintaining β-cell identity in individuals with obesity and T2D.

Another important observation of our study is that B7-H4 regulates insulin secretion by modulating cholesterol metabolism in β cells. The insufficiency of serum insulin in B7-H4 cKO mice after glucose injection was partly due to decreased β-cell mass. However, when we took the same number of islets *ex vivo* and stimulated them with glucose, insulin secretion from the islets of B7-H4 cKO mice decreased significantly, indicating that the defect in insulin secretion was also a crucial factor. The SEM results revealed that the vesicles in β cells from B7-H4 cKo mice were numerous but immature and further away from the plasma membrane. To investigate the molecular mechanism by which B7-H4 affects β-cell function, we focused on the 75 differential genes altered by B7-H4 KO but reversed by B7-H4 overexpression. To our surprise, these 75 genes were enriched in cholesterol metabolism. Cholesterol metabolism has been reported to be involved in the maintenance of β-cell function and insulin secretion. Excess cholesterol in β cells impairs β-cell function, causing insulin secretory vesicle enlargement and reduced glucose-stimulated secretion [[28], [29], [30], [31], [32]]. Indeed, islet cholesterol content detection revealed that the islet cholesterol content of B7-H4 cKO mice was significantly increased, whereas B7-H4 overexpression significantly reduced the islet cholesterol content. By comparing the 75 genes in mice with the DEGs in healthy humans with high and low expression of B7-H4 in the GTEx database, we found that Apof is a common gene regulated by B7-H4 in both humans and mice. We verified the downregulated protein levels in islets from B7-H4 cKO mice and upregulated levels in those from B7-H4-overexpressing mice. Moreover, LTIP (lipid transfer inhibitor protein) inhibits the activity of CETP (cholesteryl ester transfer protein) and, via this inhibition, functions to regulate cholesterol transport [53]. The expression and function of Apof in β cells are still unknown. However, other apolipoproteins, such as apolipoprotein A-I, reportedly improve pancreatic β-cell function [33] and protect them from cholesterol-induced mitochondrial damage, restoring their ability to secrete insulin [34]. The overexpression of Apof in the β cells of B7-H4 cKO mice, which have reduced Apof expression, markedly rescued the intolerance of glucose nearly to normal levels, indicating that the downregulation of Apof expression was the main cause of β-cell dysfunction caused by B7-H4 KO.

The initial cephalic phase and postprandial insulin secretion have been reported to be modulated by cytokines such as IL-β and IL-33 [[54], [55], [56]]. Here, we found that many types of cytokines and chemokine receptors were upregulated or downregulated in islets from B7-H4 cKO mice. Cytokines typically function through the downstream JAK-STAT signalling pathway. We confirmed that Stat5 plays a key role in the regulation of insulin secretion by B7-H4 and that inhibiting Stat5 can reverse impaired glucose tolerance and insulin secretion in B7-H4 cKO mice. This effect of Stat5 is achieved by regulating the concentration of islet cholesterol, and Stat5 inhibitors significantly increase the expression of Apof in islets from B7-H4 cKO mice. Stat5 has been reported to be involved in the regulation of cholesterol metabolism [35,57], although the molecular mechanism is not clear. As a transcription factor, Stat5 usually activates the expression of target genes. However, we found that the expression of Apof was upregulated after Stat5 inhibition, which may be related to the indirect role of Stat5 in acting as a cytoplasmic platform for other proteins, as reported previously [57]. Indeed, specific knockout of Stat5 in the liver can upregulate bile acid conversion genes [35].

In this study, we identified the role of B7-H4 in regulating β-cell mass and insulin processing and secretion through cholesterol metabolism in β-cell-specific B7-H4 knockout mice and HFD-fed mice with β-cell-specific B7-H4 overexpression. This is the first *in vivo* evidence showing that B7-H4 plays a physiological role in regulating β-cell mass and insulin secretion, which are modulated by Stat5 signalling. Our findings identify B7-H4 as a regulator of β-cell mass and insulin secretion, and its manipulation in β cells offers a therapeutic opportunity for T2D.

## 5 Limitations of this study

There are still some unanswered questions in our study. First, how does B7-H4 affect Stat5 signaling, and is it related to cytokines? What is the cellular source of these cytokines? Second, how does Stat5 regulate the expression of Apof? Third, although we used two kinds of Cre mice (RIP Cre and Pdx-1Cre) to exclude the effects of transgene and non-specific roles, both Ins2 and Pdx-1genes may be expressed in the hypothalamus, so the influence of B7-H4 in the hypothalamus cannot be excluded. Overall, our study elucidates, for the first time, the physiological function of B7-H4 in regulating insulin secretion through cholesterol metabolism.

## CRediT authorship contribution statement

**Fangzhen Xia:** Writing – review & editing, Writing – original draft, Visualization, Validation, Supervision, Project administration, Methodology, Investigation, Funding acquisition, Formal analysis, Data curation, Conceptualization. **Ziteng Zhang:** Writing – review & editing, Writing – original draft, Visualization, Methodology, Investigation, Formal analysis. **Zhen Qian:** Writing – review & editing, Writing – original draft, Visualization, Validation, Methodology, Investigation, Formal analysis. **Xiaoyu Fang:** Writing – review & editing, Visualization, Formal analysis. **Junxue Wang:** Writing – review & editing. **Yan Wang:** Writing – review & editing, Visualization. **Guoting Sun:** Writing – review & editing. **Yuefeng Yu:** Writing – review & editing, Visualization. **Ninjian Wang:** Writing – review & editing. **Junke Zhen:** Writing – review & editing, Validation, Methodology, Investigation, Data curation, Conceptualization. **Yan Liu:** Writing – review & editing, Validation, Methodology, Investigation, Data curation, Conceptualization. **Yingli Lu:** Writing – review & editing, Validation, Supervision, Project administration, Methodology, Investigation, Funding acquisition, Data curation, Conceptualization.

## Declaration of competing interest

The authors declare no competing interests.

## Data Availability

Data will be made available on request.

## Appendix A Supplementary data

Multimedia component 1 Multimedia component 1

sFigure 1 **B7-H4 expression is evaluated in diabetic pancreatic β cells.** (A) Western blot analysis of B7-H4 expression in different tissue of mice. (B) Representative images of pancreatic islet immunofluorescence staining in the indicated groups. Red, B7-H4; Blue, DAPI; Green, Insulin, Glucagon, CD31 or F4/80 in respective image. (C) B7-H4 mRNA expression of MIN6 cell incubated in the medium with various concentrations of palmitate for 48h. n = 12 per group. Data are shown as mean ± SEM. one-way ANOVA with Bonferroni's multiple comparison was used in (C). Three independent experiments were performed. sFigure 1

sFigure 2 **B7-H4 knock out in pancreatic β-cells damages glucose disposal and insulin secretion.** (A) Generation process of β-cell–specific B7-H4 knockout (cKO) mice. (B) Representative genotype results of offspring by PCR. (C) B7-H4 mRNA expression in the indicated groups. Flox, n = 3; cKO, n = 8. (D) Western blot of B7-H4 protein expression in the indicated groups. n = 3 per group. (E-J) Food intake, serum cholesterol, serum triglyceride, fasted blood glucose, random blood glucose, and fasted serum glucagon of mice in the indicated groups. n = 6–13 per group, Data are shown as mean ± SEM. ∗∗∗: *P* < 0.001; *n.s.*: not significant. Unpaired two-tailed Student's t-test was used in (E)-(J). Three independent experiments were performed. sFigure 2

sFigure 3 **Cre transgene has limited effects in glucose disposal.** (A-E) GTT results of 4 weeks (A), 8 weeks (B and E), 12 weeks (C) and 16 weeks (D) mice in the indicated groups suggesting that the impaired glucose tolerance in B7-H4 cKO mice is mainly caused by the specific knockout B7-H4 in β cells. n = 5–8 per group. Data are shown as mean ± SEM. Three independent experiments were performed. sFigure 3

sFigure 4 **B7-H4 overexpression in pancreatic β-cells improves glucose disposal and insulin secretion.** (A) Representative fluorescence images of pancreatic islet isolated from mice after 5 weeks of virus infusion. (B) mRNA expression of B7-H4 in islet isolated from mice after 5 weeks of virus infusion. n = 9–18 per group. (C) Protein expression of B7-H4 in islet isolated from mice after 5 weeks of virus infusion. n = 3 per group. Ctrl: Flox mice injected with AAV2/8-Ctrl; OE: Flox mice injected with AAV2/8-Ins2-B7H4. (D and E) ITT results after 4 weeks of virus infusion in the indicated groups. n = 18 per group. Data are shown as mean ± SEM. ∗∗: *P* < 0.01; *n.s.*: not significant. Unpaired two-tailed Student's t-test was used in (B) and (E). Three independent experiments were performed. sFigure 4

sFigure 5 **RNA-seq on islets indicates B7-H4 changed the expression of β cell function and insulin secretion markers** (A and B) GO analysis of differentially expressed genes in Flox (n = 3) and cKO (n = 3), or in AAV2/8-Ins2-B7H4 (n = 3) and AAV2/8-Ctrl (n = 3) showed that cell adhesin, potassium ion transport, cholesterol efflux which were downregulated in B7-H4 cKO mice and upregulated in B7-H4 overexpression mice. sFigure 5

sFigure 6 **B7-H4 maintains β cell function and insulin secretion by regulating islet cholesterol metabolism.** (A and B) KEGG and GO analysis of the identified differential expressed genes showed that DEGs in high and low B7-H4 enriched in cytokine–cytokine receptor interaction, lipid and atherosclerosis and chemokine signaling pathway. (B) Western blot of Apof protein expression in the indicated groups. n = 3 per group. Ctrl: Flox mice injected with AAV2/8-eGFP; OE: Flox mice injected with AAV2/8-Apof-3xFlag-eGFP (D and E) ITT results of mice in the indicated groups. n = 4–5 per group. Data are shown as mean ± SEM. ∗: *P* < 0.05; *n.s.*: not significant. Unpaired two-tailed Student's t-test was used in (E). Three independent experiments were performed. sFigure 6
