# Supplementary material for: The immune checkpoint molecule B7-H4 regulates β-cell mass and insulin secretion by modulating cholesterol metabolism through Stat5 signalling
Source: Mol Metab. 2024 Nov 19;91:102069. doi: 10.1016/j.molmet.2024.102069 (PMC11636127; doi:10.1016/j.molmet.2024.102069)
Supplement: Multimedia component 1 [file mmc1.docx]

| **Supplemental table**  **Table S1. PCR primer sequences** | |
| --- | --- |
| **Primer** | **sequence (5'-3')** |
| mB7H4-F1 | GGCAATGCTTCCCTGAGACT |
| mB7H4-R1 | TTGGTCGACTTGAGATGCCC |
| mB7H4-F2 | TCAGGCAAGCACTTCATCAC |
| mB7H4-R2 | GGTCGTCTTTGCCTTCTTTG |
| mIns1-F1 | GCTCTCTACCTGGTGTGTGG |
| mIns1-R1 | AACGCCAAGGTCTGAAGGTC |
| mIns2-F1 | TGCTATCCTCAACCCAGCCTA |
| mIns2-R1 | CTCCAGTTGTGCCACTTGT |
| mCasepase3-F1 | TGGCTTGCCAGAAGATACCG |
| mCasepase3-R1 | CCGTTGCCACCTTCCTGTTA |
| mCcl2-F1 | TTAAAAACCTGGATCGGAACCAA |
| mCcl2-R1 | GCATTAGCTTCAGATTTACGGGT |
| mCcl17-F1 | ACCCTGGCTTTGCTTAATAGTG |
| mCcl17-R1 | GAAGCCCATAGAGCATCCCC |
| mCcl22-F1 | AACCTTCTTGCTCCTCTGGA |
| mCcl22-R1 | AAGCCCTTTGTGGTCCCATA |
| mCcl24-F1 | ATTCTGTGACCATCCCCTCAT |
| mCcl24-R1 | TGTATGTGCCTCTGAACCCAC |
| mCcl27-F1 | CCTCCCGCTGTTACTGTTG |
| mCcl27-R1 | TTCCATGTGGACAATCCTCCT |
| mCcr2-F1 | ATCCACGGCATACTATCAACATC |
| mCcr2-R1 | CAAGGCTCACCATCATCGTAG |
| mCcr4-F1 | GGAAGGTATCAAGGCATTTGGG |
| mCcr4-R1 | GTACACGTCCGTCATGGACTT |
| mCcr5-F1 | ATGGATTTTCAAGGGTCAGTTCC |
| mCcr5-R1 | CTGAGCCGCAATTTGTTTCAC |
| mCcr3-F1 | TGGGCAACATGATGGTTGTG |
| mCcr3-R1 | GCTGTCTTGAGACTCATGGA |
| mCcr10-F1 | GGACTTTACTCCGGGTACGAT |
| mCcr10-R1 | CAGGGAGACACTGGGTTGGA |
| mIl1-F1 | GCAACTGTTCCTGAACTCAACT |
| mIl1-R1 | ATCTTTTGGGGTCCGTCAACT |
| mIl4-F1 | GGTCTCAACCCCCAGCTAGT |
| mIl4-R1 | GCCGATGATCTCTCTCAAGTGAT |
| mIl6-F1 | TAGTCCTTCCTACCCCAATTTCC |
| mIl6-R1 | TTGGTCCTTAGCCACTCCTTC |
| mIl10-F1 | GCTCTTACTGACTGGCATGAG |
| mIl10-R1 | CGCAGCTCTAGGAGCATGTG |
| mRab3a-F1 | TCCCAGTCCCCTGGAAAAAC |
| mRab3a-R1 | GCTCCTCCTTTAGGAACTCGG |
| mRab27a-F1 | TCGGATGGAGATTACGATTACCT |
| mRab27a-R1 | TTTTCCCTGAAATCAATGCCCA |
| mSnap25-F1 | TGCACGCGGTCAGAGAAAG |
| mSnap25-R1 | TCGTCGCCTGGCATTAAGC |
| mVamp2-F1 | GCTGGATGACCGTGCAGAT |
| mVamp2-R1 | GATGGCGCAGATCACTCCC |
| mBcl2-F1 | GGAAGGTAGTGTGTGTGG |
| mBcl2-R1 | ACTCCACTCTCTGGGTTCTTGG |
| mBax-F1 | ACAGATCATGAAGACAGGGG |
| mBax-R1 | CAAAGTAGAAGAGGGCAACC |
| mIns2Cre-F1 | ACTCCAAGTGGAGGCTGAGA |
| mIns2Cre-R1 | TCCTTCCACAAACCCATAGC |
| mloxp-F1 | CCTAGATGGGAGAAATGAAAGGGT |
| mloxp-R1 | GCAATAGAGCATGTGCTTAGAACC |
| mPdxCre-F1 | CCTGGACTACATCTTGAGTTGC |
| mPdxCre-R1 | AGGCAAATTTTGGTGTACGG |
| mβ-Actin-F1 | CAGCCTTCCTTCTTGGGTATG |
| mβ-Actin-R1 | GGCATAGAGGTCTTTACGGATG |

| **TableS2. KEY RESOURCES TABLE** |  |  |
| --- | --- | --- |
| **REAGENT or RESOURCE** | **SOURCE** | **IDENTIFIER** |
| **Antibodies** |  |  |
| Goat Anti-Mouse B7-h4 Polyclonal antibody, Unconjugated | R and D Systems | Cat# AF2154, RRID: AB_2216146 |
| B7H4 antibody [EP1165] | Abcam | Cat# ab108336, RRID: AB_10866162 |
| Anti-ApoF antibody | Abcam | Cat# ab231585, RRID:NA |
| Stat5 antibody | Cell Signaling Technology | Cat# 94205, RRID: AB_2737403 |
| Phospho-Stat5 (Tyr694) (D47E7) XP Rabbit mAb | Cell Signaling Technology | Cat# 4322, RRID: AB_10544692 |
| GAPDH antibody | Proteintech | Cat# 60004-1-Ig, RRID: AB_2107436 |
| Beta Actin antibody | Proteintech | Cat# 66009-1-Ig, RRID: AB_2687938 |
| Recombinant Anti-Iba1 antibody | Abcam | Cat# ab178846, RRID: AB_2636859 |
| Anti-Insulin Antibody [EPR17359] | Abcam | Cat# ab181547, RRID: AB_2716761 |
| Anti-Glucagon Antibody, Unconjugated | Cell Signaling Technology | Cat# 2760, RRID: AB_659831 |
| Cleaved Caspase-3 (Asp175) | Cell Signaling Technology | Cat# 9661, RRID: AB_2341188 |
| F4/80 antibody [CI:A3-1] | Abcam | Cat# ab6640, RRID: AB_1140040 |
| Lamin B1 antibody | Proteintech | Cat# 12987-1-AP, RRID: AB_2136290 |
| Alpha Tubulin antibody | Proteintech | Cat# HRP-66031, RRID: AB_2687491 |
| Recombinant Anti-CD31 antibody [EPR17259] | Abcam | Cat# ab182981, RRID: AB_2920881 |
| Goat Anti-Rabbit IgG H&L (Alexa Fluor® 488) | Abcam | Cat# ab150077, RRID: AB_2630356 |
| Goat Anti-Rabbit IgG - H&L Polyclonal Antibody, Cy3 Conjugated | Abcam | Cat# ab6939, RRID: AB_955021 |
| **Bacterial and virus strains** |  |  |
| AAV2/8-Ins2-B7H4 | Hanbio | NA |
| AAV2/8-Ctrl | Hanbio | NA |
| AAV2/8-Apof-3xFlag-eGFP | Genomeditech | NA |
| AAV2/8-eGFP | Genomeditech | NA |
| **Chemicals, peptides, and recombinant proteins** |  |  |
| Pimozide | Selleck | Cat#S4358 |
| Human insulin (Novolin R) | Novo Nordisk A/S | NA |
| Collagenase P | Roche | Cat#33768624 |
| DNAse I | Roche | Cat#1014159001 |
| HBSS | Biosharp | Cat#BL559A |
| Bovine serum albumin | Sigma-Aldrich | Cat#10711454001 |
| DMEM | Gibco | Cat#11885084 |
| DMEM | Gibco | Cat#11965092 |
| Penicillin | Gibco | Cat#15140-122 |
| Fetal bovine serum | Gibco | Cat#A3160801 |
| PrimeScript™ RT Master Mix | TAKARA | Cat#RR036A |
| HEPES | Gibco | Cat#15630-080 |
| L-glutamine | Gibco | Cat#25030-081 |
| Sodium pyruvate | Gibco | Cat#11360-070 |
| β-Mercaptoethanol | Sigma-Aldrich | Cat#444203 |
| Palmitate | Sigma-Aldrich | Cat#P9767 |
| Recombinant IL-1β | Peprotech | Cat#400-01B |
| **Critical commercial assays** |  |  |
| Highly Sensitive Mouse Insulin ELISA kit | EZassy | Cat#MS100 |
| Cytoplasmic and Nuclear Protein Extraction Kit | Solarbio | Cat#R0050 |
| Triglyceride assay kit | Nanjing Jiancheng | Cat#A110-1-1 |
| LabAssay cholesterol kit | Wako | Cat#KBR0082 |
| Mouse Glucogon EIL Kit | RayBio | Cat#EIAM-GLU-1 |
| Wide Filed Mouse Insulin ELISA kit | EZassy | Cat#MS300 |
| BCA Kit | Solarbio | Cat#PC0020 |
| RNeasy Mini Kit | QIAGEN | Cat#74104 |
| RNAiso Plus | Takara | Cat#9108 |
| **Experimental models: Cell lines** |  |  |
| MIN6 cell | ,QuiCell Biotechnology | Cat#QuiCell-M308 |
| **Experimental models: Organisms/strains** |  |  |
| Mouse: B7-H4 fl/fl | Cyagen Biosciences | Cat#S-CKO-08415 |
| Mouse: Pdx-Cre | Jackson Laboratory | Cat#C001033 |
| Mouse: RIP-Cre | Jackson Laboratory | Cat#C001002 |
| Mouse: Rip-Cre; B7-H4 fl/fl ; B7-H4 cKO | NA | NA |
| Mouse: Pdx-Cre; B7-H4 fl/fl; PB7-H4 cKO | NA | NA |
| **Software and algorithms** |  |  |
| NIS-Elements software | Nikon | https://industry.nikon.com/ |
| RStudio 4.2.1 | Posit | https://posit.co/download/rstudio-desktop/ |
| ImageJ | National Institutes of Health | https://imagej.net/ij/ |
| Cellpose | NA | https://www.cellpose.org/ |
| GraphPad Prism | GraphPad Software | https://www.graphpad.com/ |
