# Supplementary figures and images for: The immune checkpoint molecule B7-H4 regulates β-cell mass and insulin secretion by modulating cholesterol metabolism through Stat5 signalling
Source: Mol Metab. 2024 Nov 19;91:102069. doi: 10.1016/j.molmet.2024.102069 (PMC11636127; doi:10.1016/j.molmet.2024.102069)

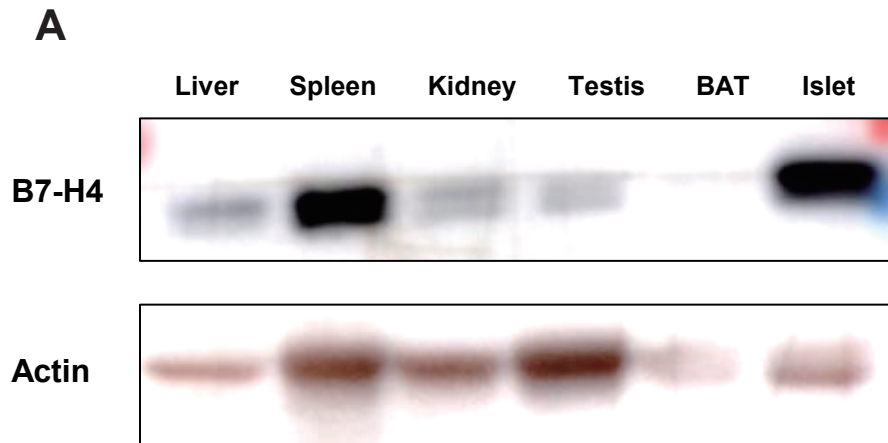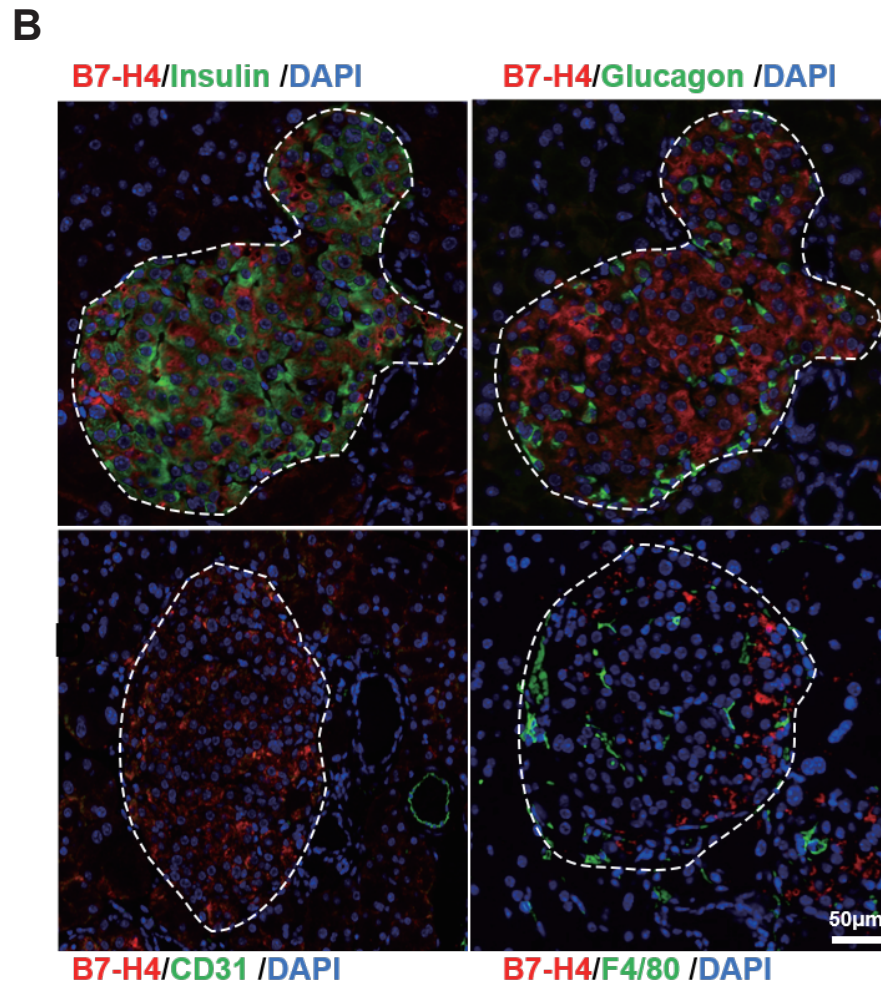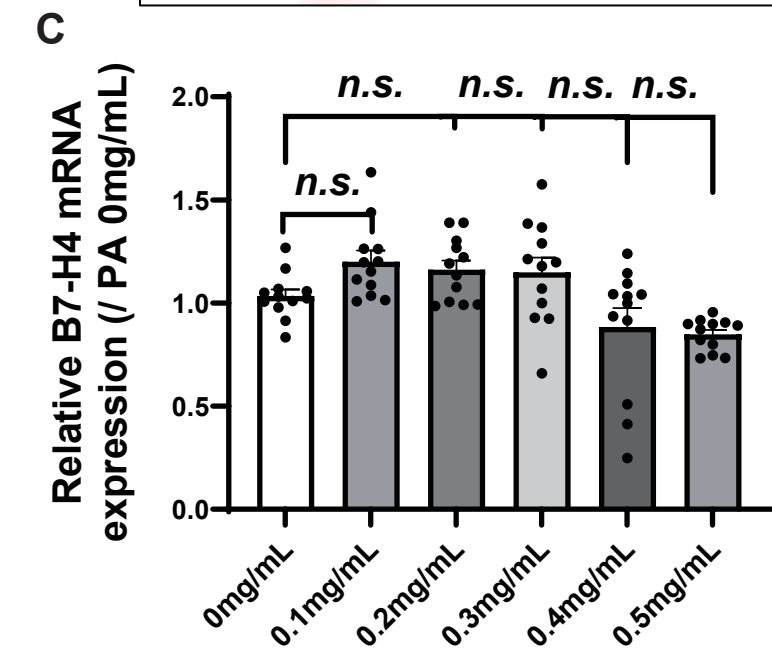

Supplement: sFigure 1 — B7-H4 expression is evaluated in diabetic pancreatic β cells. (A) Western blot analysis of B7-H4 expression in different tissue of mice. (B) Representative images of pancreatic islet immunofluorescence staining in the indicated groups. Red, B7-H4; Blue, DAPI; Green, Insulin, Glucagon, CD31 or F4/80 in respective image. (C) B7-H4 mRNA expression of MIN6 cell incubated in the medium with various concentrations of palmitate for 48h. n = 12 per group. Data are shown as mean ± SEM. one-way ANOVA with Bonferroni's multiple comparison was used in (C). Three independent experiments were performed. [file mmc2.pdf]

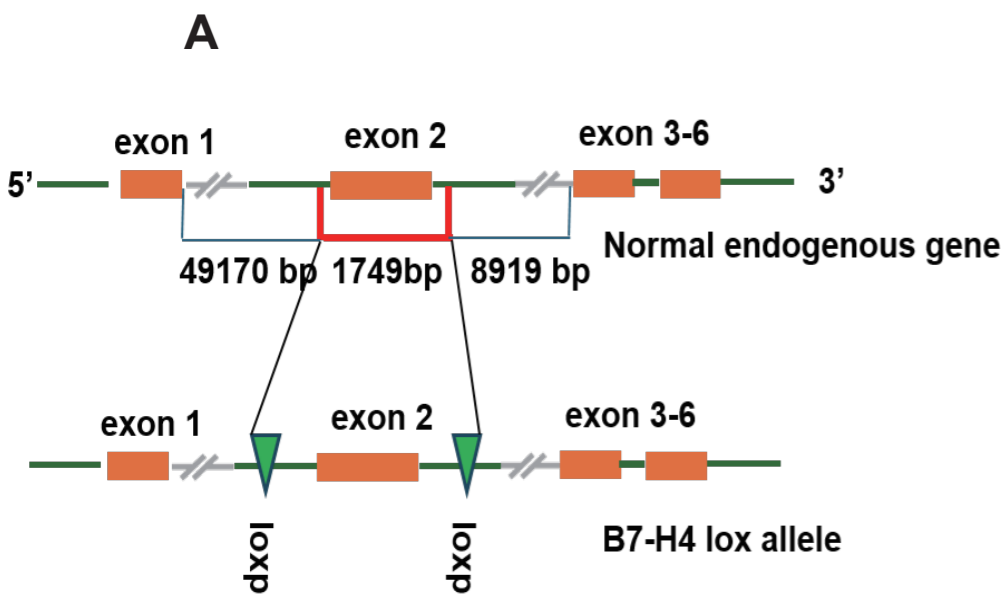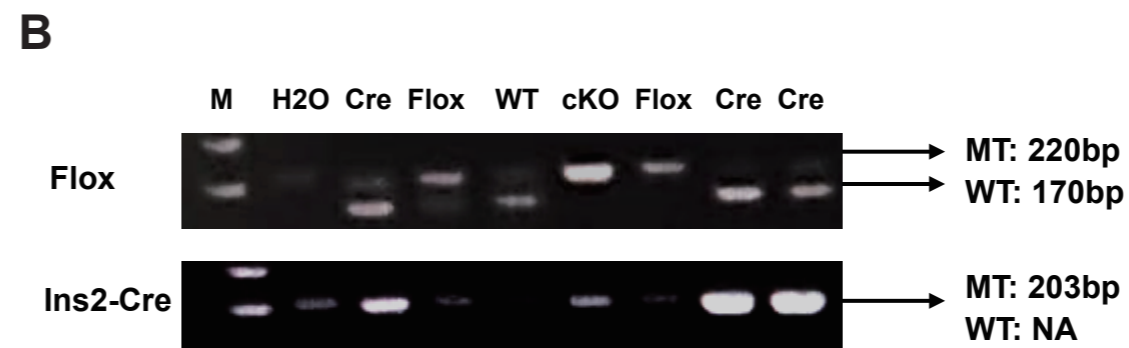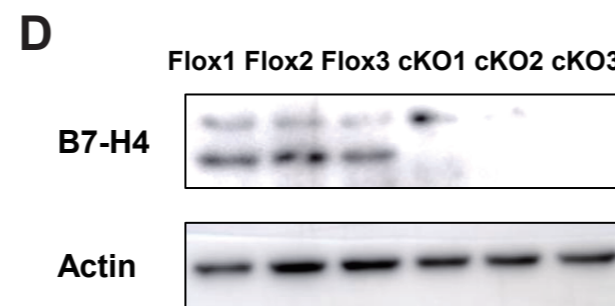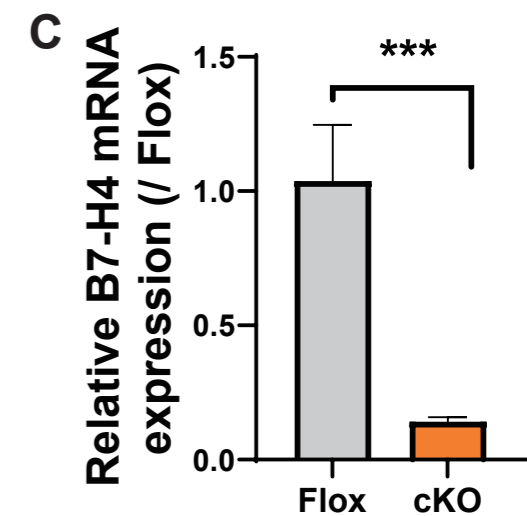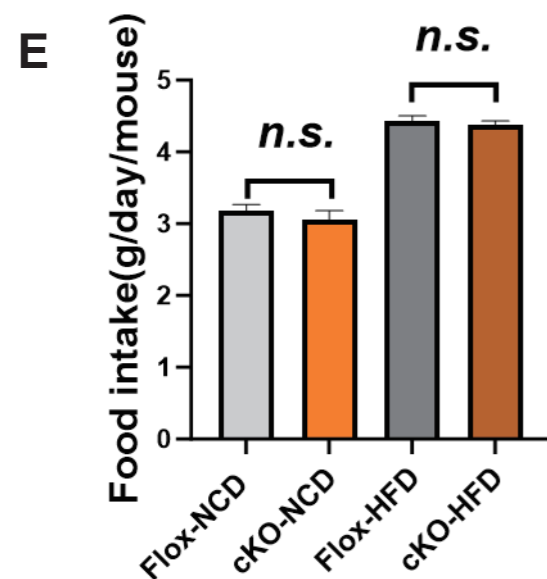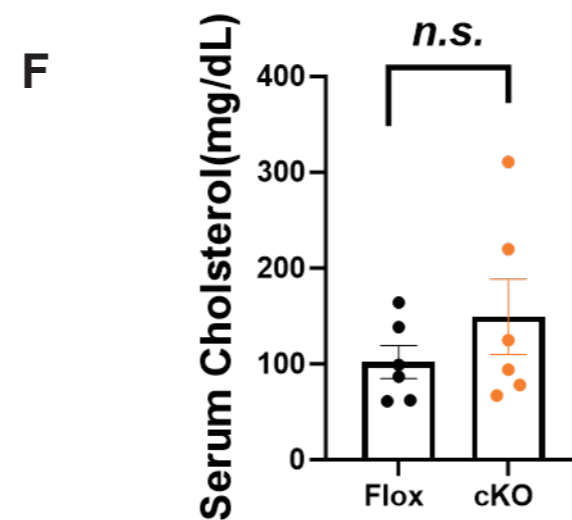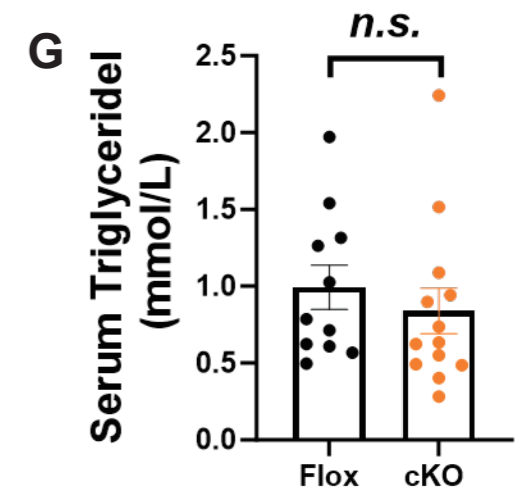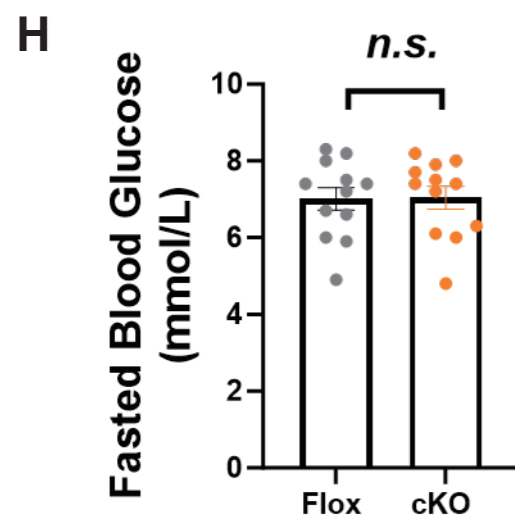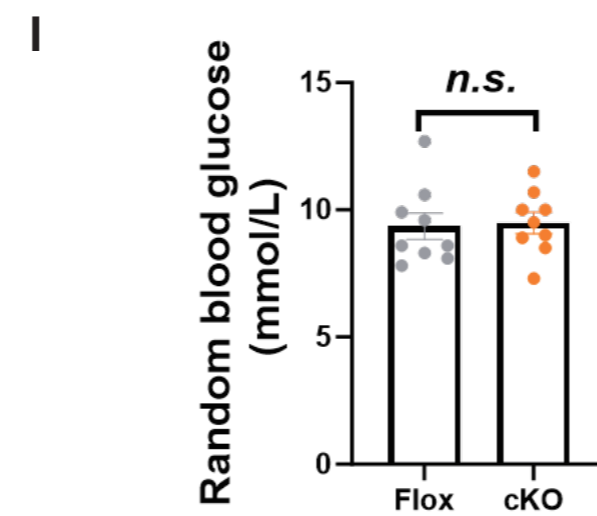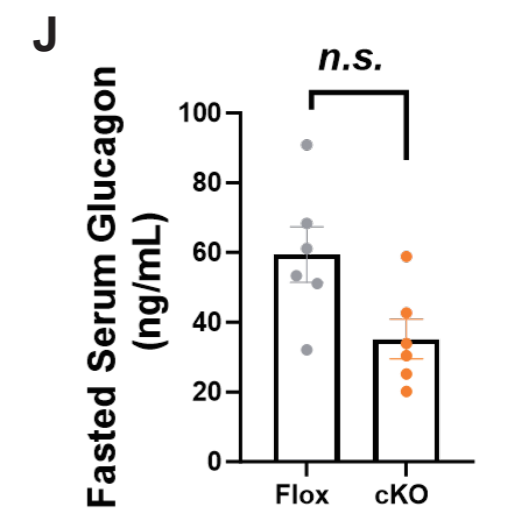

Supplement: sFigure 2 — B7-H4 knock out in pancreatic β-cells damages glucose disposal and insulin secretion. (A) Generation process of β-cell–specific B7-H4 knockout (cKO) mice. (B) Representative genotype results of offspring by PCR. (C) B7-H4 mRNA expression in the indicated groups. Flox, n = 3; cKO, n = 8. (D) Western blot of B7-H4 protein expression in the indicated groups. n = 3 per group. (E-J) Food intake, serum cholesterol, serum triglyceride, fasted blood glucose, random blood glucose, and fasted serum glucagon of mice in the indicated groups. n = 6–13 per group, Data are shown as mean ± SEM. ∗∗∗: P < 0.001; n.s.: not significant. Unpaired two-tailed Student's t-test was used in (E)-(J). Three independent experiments were performed. [file mmc3.pdf]

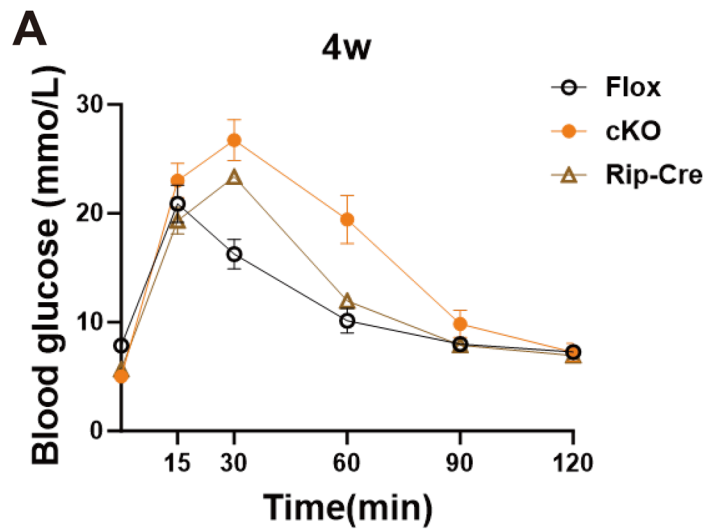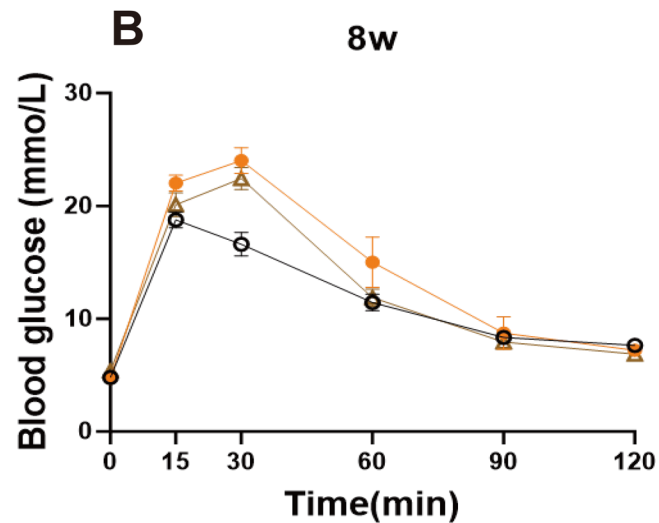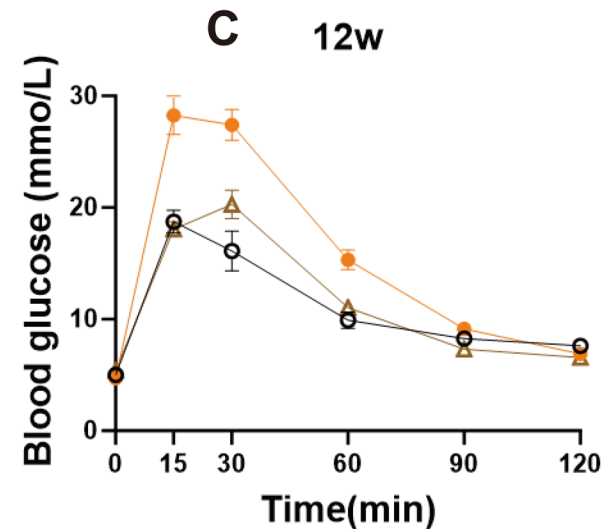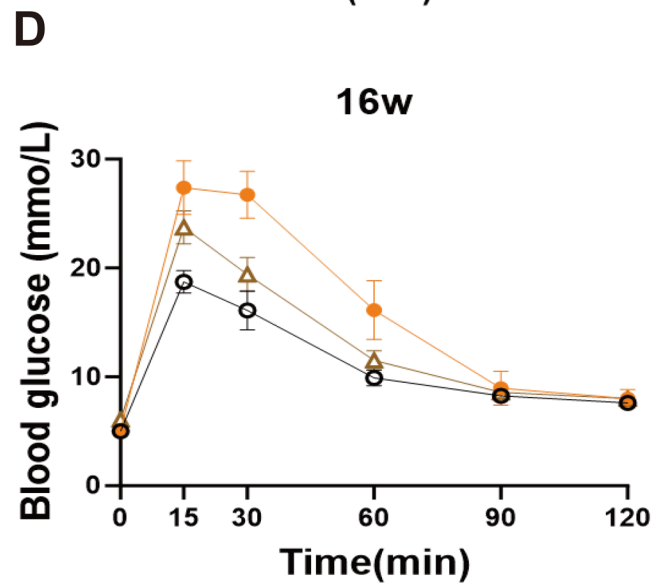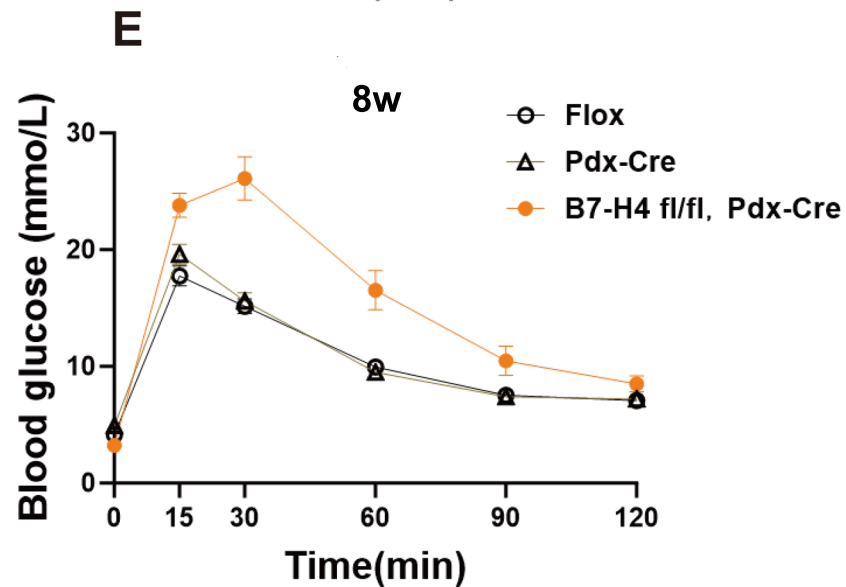

Supplement: sFigure 3 — Cre transgene has limited effects in glucose disposal. (A-E) GTT results of 4 weeks (A), 8 weeks (B and E), 12 weeks (C) and 16 weeks (D) mice in the indicated groups suggesting that the impaired glucose tolerance in B7-H4 cKO mice is mainly caused by the specific knockout B7-H4 in β cells. n = 5–8 per group. Data are shown as mean ± SEM. Three independent experiments were performed. [file mmc4.pdf]

**A**

AAV2/8-Ctrl

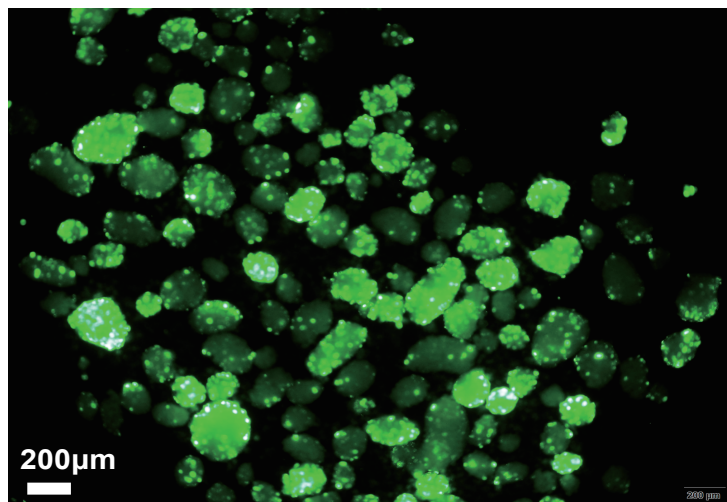

AAV2/8-Ins2-B7H4

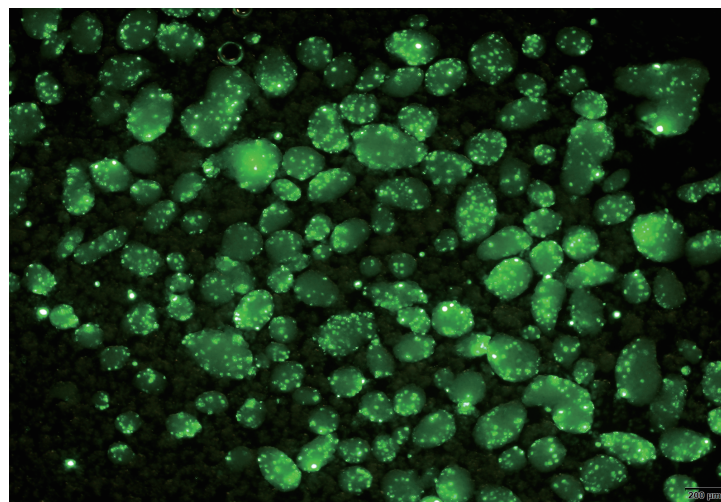**B**

Relative B7-H4 mRNA expression (/ Ctrl)

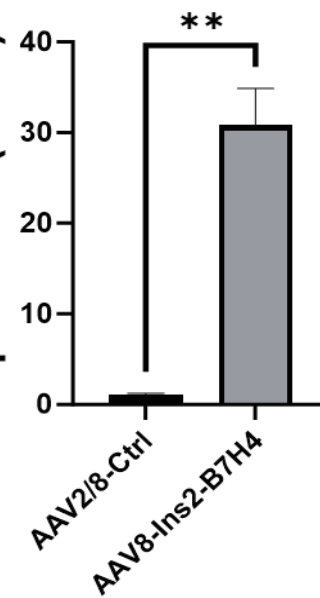**C**

Ctrl1 Ctrl2 Ctrl3 OE1 OE2 OE3

B7-H4

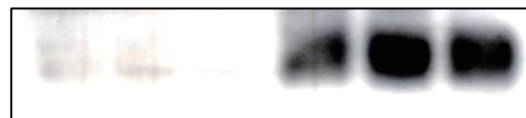

Actin

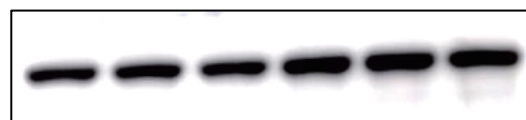**D**

IPITT

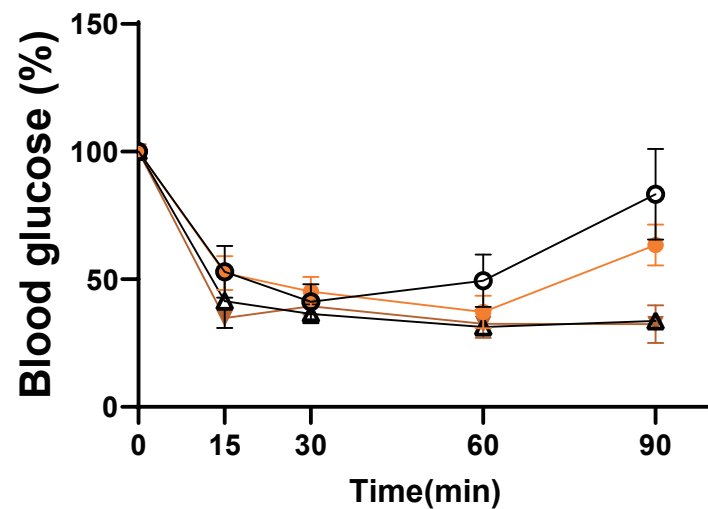**E**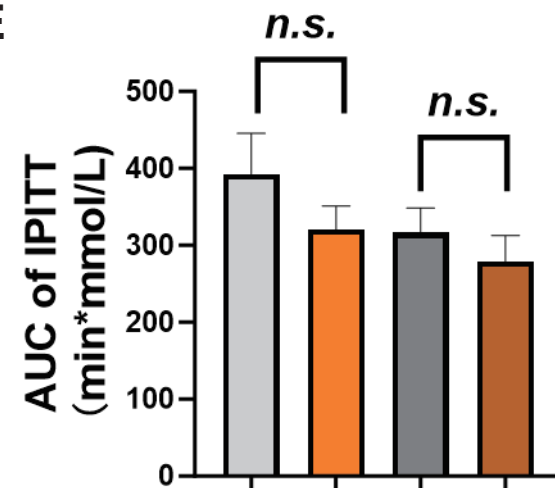

Supplement: sFigure 4 — B7-H4 overexpression in pancreatic β-cells improves glucose disposal and insulin secretion. (A) Representative fluorescence images of pancreatic islet isolated from mice after 5 weeks of virus infusion. (B) mRNA expression of B7-H4 in islet isolated from mice after 5 weeks of virus infusion. n = 9–18 per group. (C) Protein expression of B7-H4 in islet isolated from mice after 5 weeks of virus infusion. n = 3 per group. Ctrl: Flox mice injected with AAV2/8-Ctrl; OE: Flox mice injected with AAV2/8-Ins2-B7H4. (D and E) ITT results after 4 weeks of virus infusion in the indicated groups. n = 18 per group. Data are shown as mean ± SEM. ∗∗: P < 0.01; n.s.: not significant. Unpaired two-tailed Student's t-test was used in (B) and (E). Three independent experiments were performed. [file mmc5.pdf]

A

cKO-vs-Flox(Down)  
Top 30 GO Term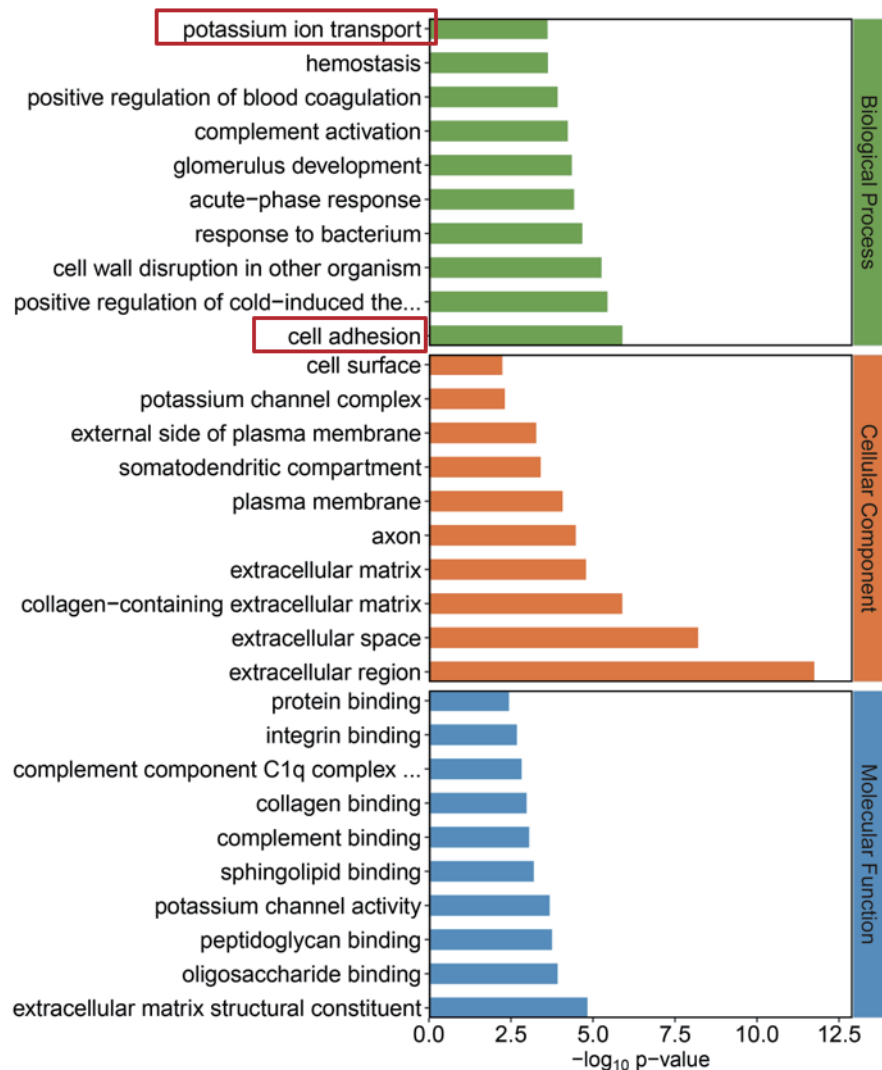

B

AAV8-B7H4-vs-AAV8-Ctrl(Up)  
Top 30 GO Term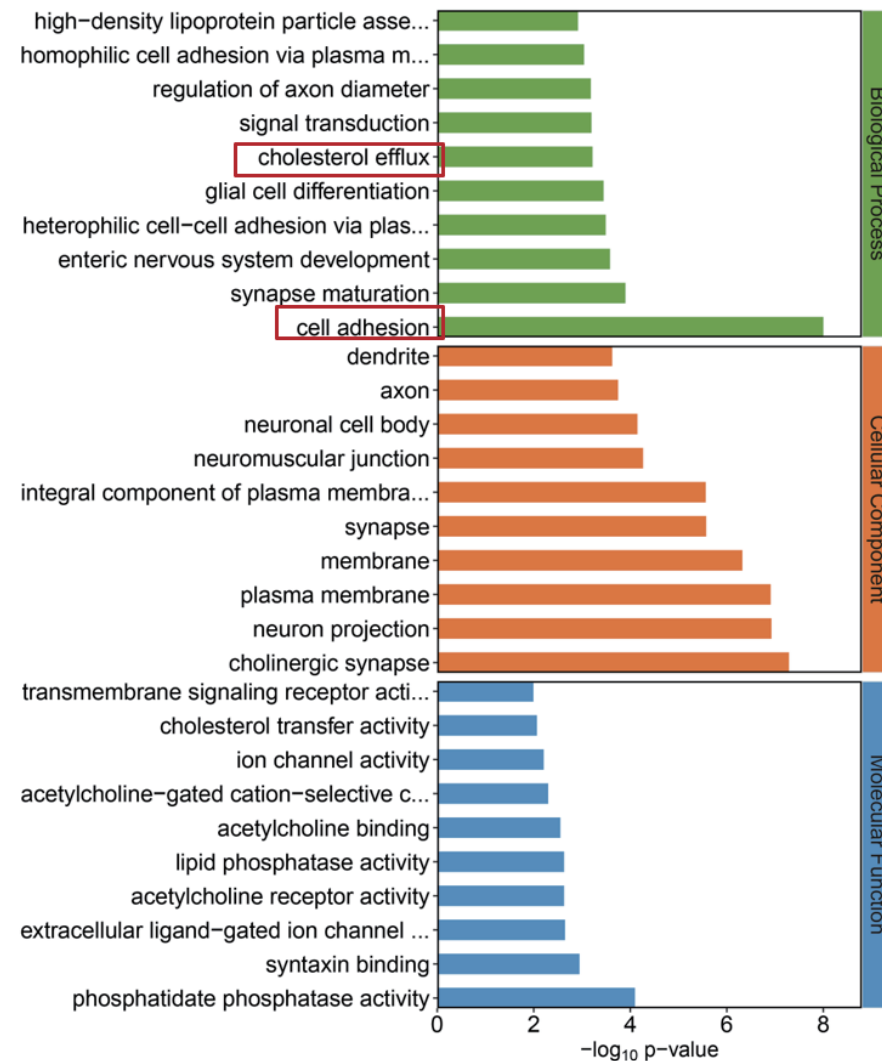

Supplement: sFigure 5 — RNA-seq on islets indicates B7-H4 changed the expression of β cell function and insulin secretion markers (A and B) GO analysis of differentially expressed genes in Flox (n = 3) and cKO (n = 3), or in AAV2/8-Ins2-B7H4 (n = 3) and AAV2/8-Ctrl (n = 3) showed that cell adhesin, potassium ion transport, cholesterol efflux which were downregulated in B7-H4 cKO mice and upregulated in B7-H4 overexpression mice. [file mmc6.pdf]

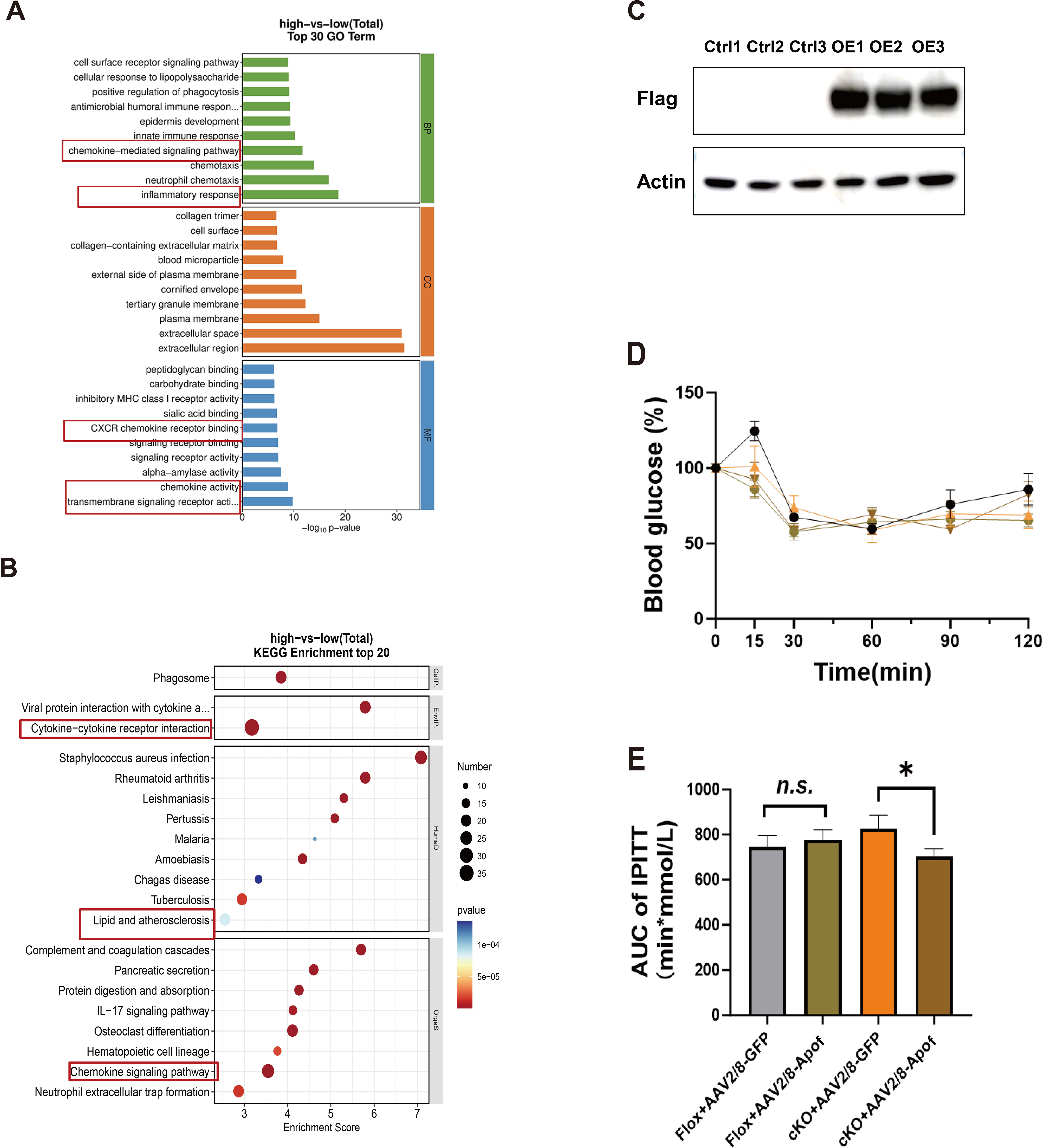

Supplement: sFigure 6 — B7-H4 maintains β cell function and insulin secretion by regulating islet cholesterol metabolism. (A and B) KEGG and GO analysis of the identified differential expressed genes showed that DEGs in high and low B7-H4 enriched in cytokine–cytokine receptor interaction, lipid and atherosclerosis and chemokine signaling pathway. (B) Western blot of Apof protein expression in the indicated groups. n = 3 per group. Ctrl: Flox mice injected with AAV2/8-eGFP; OE: Flox mice injected with AAV2/8-Apof-3xFlag-eGFP (D and E) ITT results of mice in the indicated groups. n = 4–5 per group. Data are shown as mean ± SEM. ∗: P < 0.05; n.s.: not significant. Unpaired two-tailed Student's t-test was used in (E). Three independent experiments were performed. [file figs1.jpg]
